# Novelty of Bioengineered Iron Nanoparticles in Nanocoated Surgical Cotton: A Green Chemistry

**DOI:** 10.1155/2019/9825969

**Published:** 2019-02-03

**Authors:** Bhavika Turakhia, Saujanya Chikkala, Sejal Shah

**Affiliations:** Department of Microbiology, School of Science, RK University, Rajkot 360020, Gujarat, India

## Abstract

The current focus of nanotechnology is to develop environmentally safe methodologies for the formulation of nanoparticles. The phytochemistry of *Zingiber officinale* inspired us to utilize it for the synthesis of iron nanoparticles. GC-MS analysis revealed the phytochemical profile of ginger. Out of 20 different chemicals, gingerol was found to be the most potent phytochemical with a retention time of 40.48 min. The present study reports a rapid synthesis method for the formation of iron nanoparticles and its potential efficacy as an antibacterial agent and an antioxidant. Because of its antibacterial property, ginger extract was used to coat surgical cotton. Synthesized ginger root iron nanoparticles (GR-FeNPs) were characterized by UV-visible spectroscopy, Fourier-transform infrared spectroscopy (FT-IR), X-ray diffraction analysis, and particle size analysis. XRD confirmed the crystalline structure of iron oxide nanoparticles as it showed the crystal plane (2 2 0), (3 1 1), (2 2 2), and (4 0 0). The particle size analyzer (PSA) showed the average size of the particles, 56.2 nm. The antimicrobial activity of the FeNPs was tested against different Gram-positive and Gram-negative bacteria. *E. coli* showed maximum inhibition as compared with the other organisms. Antioxidant activity proved the maximum rate of free radicals at 160 *µ*g/mL produced by nanoparticles. In addition, the antimicrobial activity of nanocoated surgical cotton was evaluated on the first day and 30^th^ day after coating, which clearly showed excellent growth inhibition of organisms, setting a new path in the field of medical microbiology. Hence, iron-nanocoated surgical cotton synthesized using green chemistry, which is antimicrobial and cost effective, might be economically helpful and provide insights to the medical field, replacing conventional wound healing treatments, for better prognosis.

## 1. Introduction

In the modern era, it is crucial to find a substitute for conventional antibiotics because of the emergence of new multidrug-resistant bacterial strains that are able to form biofilms, decreasing the action of antibiotics [1]. The recent advancements in the field of nanotechnology include the preparation of nanoparticles of specific size and shape that exhibit antimicrobial properties. The antimicrobial activity of the nanoparticles can be determined by the size, physico-chemical properties, and surface area-to-volume ratio [2–5]. It is reported that nanoparticles of smaller size tend to exhibit excellent antimicrobial activity. Various polyphenols and antioxidants present in the *Z. officinale* root play an important role in the field of medicine, and interaction of these with the metallic surface of the nanoparticles exhibits a possible antioxidant activity [4]. Because of the growing concerns of society regarding health issues, consumers are paying attention before they pick any product. That is the reason for the demand of antimicrobial agents in the market. The essential oil of *Z. officinale* has antimicrobial, antioxidant, antifungal, insecticidal, and anti-inflammatory properties [5]. It created a special interest for choosing ginger as a plant for the preparation of iron nanoparticles. Various chemical and biological methods can be used to prepare nanoparticles, but synthesis via a green approach using *Z. officinale* root extract is ecofriendly, cost effective, easy, and less hazardous [6–8].

In comparison with the previous reports, few studies have reported on the synthesis of iron oxide nanoparticles from *Z. officinale* and its antimicrobial evaluation on surgical cotton. Iron is a cost-effective alternative compared with other expensive metals reported earlier as antimicrobial agents. This study's focus is to synthesize iron nanoparticles (FeNPs) from *Z. officinale*; confirm the formation of FeNPs by different characterization methods such as UV-visible spectroscopy, Fourier transform infrared spectroscopy, and X-ray diffraction; check the bactericidal activity of FeNPs; and coat the FeNPs on surgical cotton. Because of antibiotic-resistant bacterial strains, wound dressing of patients is difficult. Thus, the study may provide insight into a new path which may be an alternative for antibiotic use in the near future.

## 2. Materials and Methods

### 2.1. Materials and Reagents

All the chemicals including ferric chloride [FeCl_3_], isopropyl alcohol, DPPH [2, 2-diphenyl-1-picrylhydrazyl], ascorbic acid, methanol, and antibiotic disks were of analytical reagent grade and used directly without any further purification. Ingredients for media preparation were from HiMedia. Ginger was collected from a local market in Gandhidham, Gujarat, India. Distilled water was used in all experiments.

### 2.2. GC-MS Analysis of *Zingiber officinale* Root Extract

The root extract of Z. officinale was analyzed using HP5 Agilent Technology 5977B (Santa Clara, US) model no. 7820A MS coupled to a 5977B equipped with a HP-5 fused silica capillary column (30 m × 0.320 mm × 0.25 *μ*m film thickness). Helium gas was used as the carrier gas. GC-MS programme was set as per the method described by Dhalani et al. [9].

### 2.3. Preparation of the Plant Extract

Ginger was collected from the local market and washed thoroughly with distilled water to eliminate dust on the surface, chopped into small pieces, sundried, and powdered. The extract was prepared by mixing 12 grams of dried ginger powder in 200 mL of isopropyl alcohol, and the mixture was stirred on a magnetic stirrer at 80°C for 1h; thereafter, the extract was filtered carefully, and the supernatant collected and stored at room temperature for further use [9].

### 2.4. Green Synthesis of Iron Nanoparticles

GR-FeNPs were synthesized by adding the equivalent extract to 0.01 M·FeCl_3_ at room temperature and constantly stirring for 10 min. Immediate appearance of black brown color showed the reduction of Fe^+3^ ions, which is the first indication of the formation of iron nanoparticles. Afterwards, the liquid was poured into large Petri plates and dried at 100°C for 24 h in a hot-air oven and cooled down the next day. The upper layer of the plate was scraped out carefully using a spatula. The fine-dried black powder of ginger iron nanoparticles was kept ready for further characterization. All nanoparticle preparations were performed according to our previous study [10, 11].

### 2.5. Characterization

#### 2.5.1. UV Visible Spectroscopy

UV visible spectroscopic analysis of the synthesized FeNPs was done by using 0.1 ml of sample diluted in 2 ml of deionized water. Absorbance was measured with the help of ABTRONICS Model No. LT-2900 in the range of 200–700 nm [12, 13].

#### 2.5.2. FT-IR Analysis (Fourier Transform Infrared Spectrophotometer)

FT-IR spectra of dried FeNPs and plant extract were determined using a Fourier transform infrared spectroscope. The synthesized nanoparticles were lyophilized and mixed with KBr pellets and further processed. An average of 9 scans were collected for each measurement with a resolution of 4 cm^−1^ at a range of 4000–650 cm^−1^ [14].

#### 2.5.3. Particle Size Analyzer (PSA)

The synthesized nanoparticles were analyzed by using a particle size analyzer, which measures the particle size by its flow through a beam of light producing a size distribution from the smallest to largest dimensions. When the particles are dissolved in water, they stay in a colloidal form and flow with a velocity that depends on their size and zeta potential (Brownian movement) [15].

#### 2.5.4. X-Ray Crystallography

The crystalline structure of the synthesized nanoparticles was analyzed by powder X-ray crystallography (XRD).

### 2.6. Antimicrobial Activity

Gram-negative bacterial strains such as *Escherichia coli* MCC 2246 and *Klebsiella pneumoniae* MCC 2716 and Gram-positive strains such as *Staphylococcus aureus* MCC 2408 and *Bacillus subtilis* MCC 2244 were cultured overnight in nutrient broth media at 37°C with continuous agitation on an orbital shaker platform at 180 rpm. Simultaneously, nutrient agar media was dispersed into sterile petri plates and incubated for 24 hours at 37°C for sterility check. The overnight broth cultures of the test organisms (*E. coli*, *K. pneumoniae*, *S. aureus*, and *B. subtilis*) were used as inoculum. Antimicrobial activity was analyzed using the agar well diffusion method. The test culture of each organism (100 *μ*l) was applied to each plate. The nanoparticles (30 *μ*g/ml) were tested against kanamycin antibiotic (30 *μ*g/disk), distilled water, ginger root extract(control), and FeCl_3_ (0.01 M). The plates were incubated for 24 hrs at 37°C [16–18]. The zone of inhibition was observed and calculated.

### 2.7. Antimicrobial Activity of Iron Nanoparticle-Coated Surgical Cotton

FeNPs (30 *µ*g/ml) were dissolved in methanol and sonicated for 10 minutes in an ultra sonicator. Dip coating is the precision- controlled immersion and withdrawal of a substrate into a reservoir of liquid for the deposition of a layer of material over it. A cotton piece of size 0.5 cm × 0.5 cm (substrate) was fixed to the head portion of the dip coater, which has a mechanical body that moves up and down. A small beaker with diluted NPs sample is placed below the body, and the dip coating process was performed three times at a particular pressure and speed. The coated cotton was carefully removed using sterile forceps, dried using an air dryer, and stored in a ziplock bag for further use. Antimicrobial activity was analyzed using the agar well diffusion method. 100 *μ*l of the test culture for each microorganism was spread using a sterile glass spreader on the nutrient agar plate and left for 10 minutes for the inoculum to get absorbed into the agar medium. A small piece of FeNPs-coated surgical cotton was placed in the middle of the plate and incubated for 48 hours, and the results noted [19, 20].

### 2.8. Antioxidant Activity of Iron Nanoparticles

Removal of free radicals using iron nanoparticles was performed using the DPPH [1, 1-diphenyl-2-picrylhydrazyl] assay. DPPH is considered a stable radical based on electron transfer (delocalization of the spare electron over the molecule as a whole) and produces a violet solution in methanol, showing a strong absorption band at 517 nm. The mixtures of different concentrations (20 *μ*g/ml, 40 *μ*g/ml, 60 *μ*g/ml, 80 *μ*g/ml, 100 *μ*g/ml, 120 *μ*g/ml, 140 *μ*g/ml, and 180 *μ*g/ml) of ginger powder, iron nanoparticles, FeCl_3_, and ascorbic acid were prepared in absolute methanol. A 0.2 mM solution of DPPH was prepared by adding 0.007 g of DPPH in 100 mL of methanol. 2 mL of DPPH solution was added to all the test tubes. After incubation of all the test tubes for 30 min, absorbance was measured at 517 nm by using a UV-vis spectrophotometer [21–23]. The experiment was performed in triplicate and % inhibition was calculated using the following equation:

(1) $$\begin{array}{c} \%\;\;\mathrm{inhibition}=\frac{\text{ OD of control}-\text{OD of sample}}{\text{OD of sample}}\times 100. \end{array}$$

## 3. Results and Discussion

### 3.1. GC-MS Analysis of *Zingiber officinale* Root Extract

**Figure 1:**
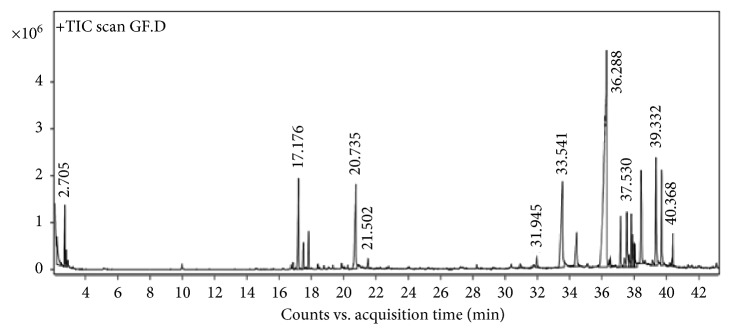
GC-MS profile of *Zingiber officinale* root extract.

The GC-MS profile of *Z. officinale* root extract is shown in Figure 1. The retention time and area (%) of each compound are given in Table 1. Out of 20 different chemical compounds, gingerol was found to be the major component with the highest retention time of 40.368 min. The presence of gingerol might be the reason for the reduction of metal ions and bactericidal activity of the nanoparticles.

### 3.2. Mechanism of FeNPs Formation from *Z. officinale* Extract

**Figure 2:**
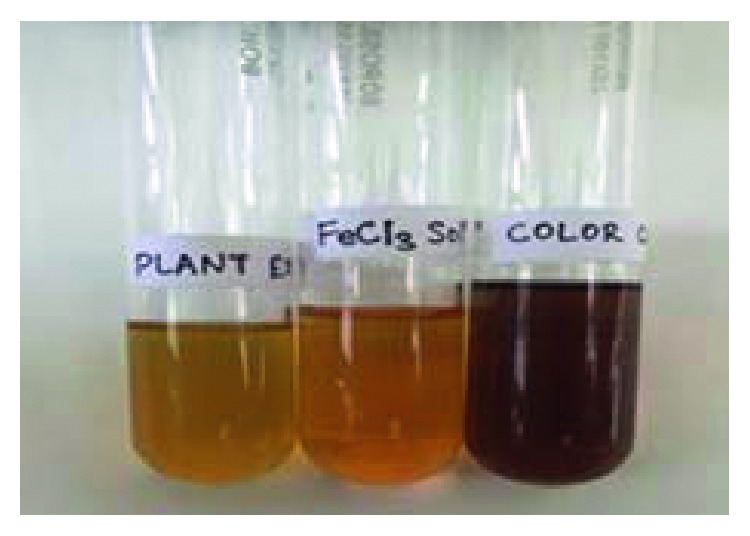
Primary indication of formation of nanoparticles—color change from yellow to brown.

The process of synthesizing nanoparticles from plant extracts has been proven to be one of the most reliable, nontoxic, and eco-friendly approaches towards green chemistry and plant biotechnology. Plant extract is mixed with 0.01 M·FeCl_3_ at a ratio of 2:3. The color change is due to the presence of polyphenols present in the plant extract which act as a reducing and capping agent, lowering the valency of Fe^+3^ to Fe^0^, as shown in Figure 2.

Earlier studies reported that the color transformation from yellow to reddish black is the primary indication of the formation of iron oxide nanoparticles [24]. The aldehyde and polyphenol groups present in the leaf extract are responsible for the reduction of ferric chloride [12, 25].

### 3.3. UV-Visible Spectroscopy (UV-Vis)

**Figure 3:**
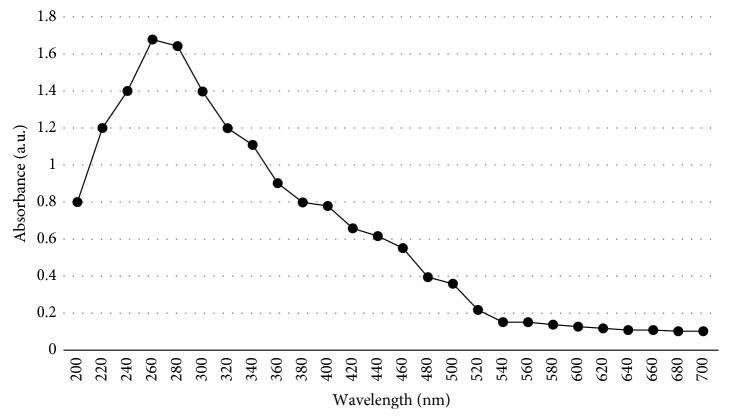
UV-visible spectroscopy analysis of synthesized GR-FeNPs.

Spectroscopy is an analytical technique concerned with the measurement of absorption of electromagnetic radiation. UV-Vis spectroscopy is one of the oldest methods in molecular spectroscopy. It refers to absorption spectroscopy in the UV-visible spectral region. This means it uses light in the visible and adjacent ranges. The absorption varies with the difference in color in the chemicals in the given samples. The UV-visible spectra of iron nanoparticles in the aqueous ginger extract are shown in Figure 3. The absorption peak at a wavelength between 200 and 260 nm indicates the formation of iron nanoparticles (Figure 3). The sharp and intense peak is attributed to the uniform size of the particles [26, 27].

### 3.4. FT-IR Analysis

**Figure 4:**
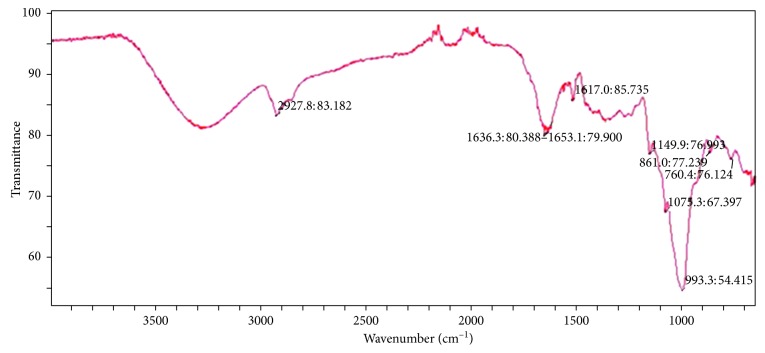
FT-IR analysis of GR-FeNPs.

FT-IR analysis was carried out to evaluate the possible interaction between the biomolecules and Fe^3+^ during the biogenic reduction reaction. The FT-IR data for FeNPs containing *Z. officinale* root extract are given in Table 2. The bond stretching at 2927.8 cm^−1^ is attributed to the C-H bond, 1638.3 cm^−1^ to the C=O bond, 1517 cm^−1^ to the C-C amide group at 861 cm^−1^, and C-N to 1075.3 cm^−1^ and 760 cm^−1^, which was found to be very close to 688 cm^−1^, which was attributed to the presence of zero valent FeNPs as shown in Figure 4. We can observe the FT-IR data of FeNPs with a plant sample [Figure 4] by analyzing the shift in bond stretching of the C-H bond from 2922 cm^−1^ to 2927 cm^−1^, C-C bond from 1514 cm^−1^ to 1517 cm^−1^, and C-N bond from 1037 cm^−1^ to 1075.5 cm^−1^. This shift in bond stretching indicates the presence of FeNPs [28–30].

### 3.5. Particle Size Analysis

**Figure 5:**
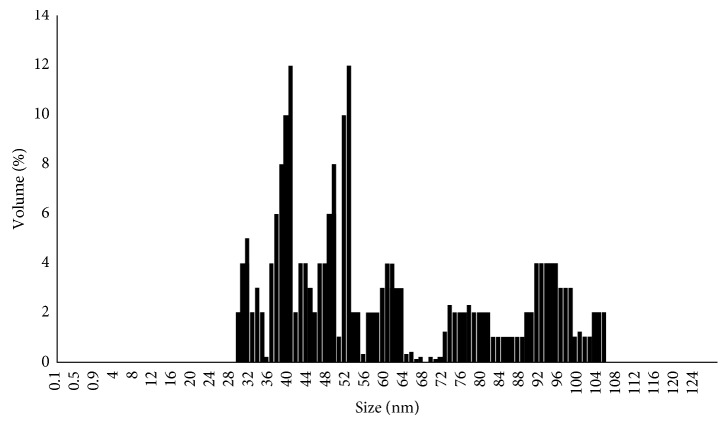
Particle size analysis of GR-FeNPs.

It is evident from the particle size analysis that smaller nanoparticles below 100 nm were synthesized, which might have agglomerated and resulted in larger nanoparticles. Furthermore, because of the magnetic property, the particles might have agglomerated, producing larger dimensions as depicted in Figure 5.

### 3.6. X-Ray Crystallography

**Figure 6:**
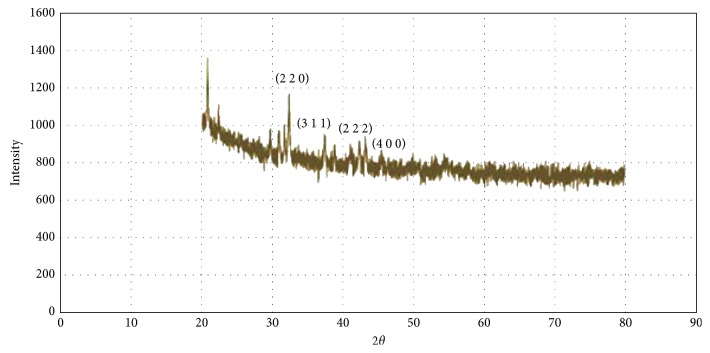
Powder X-ray diffraction pattern of GR-FeNPs.

To confirm the crystal structure of synthesized FeNPs, powder crystallography is an effective tool.  Figure 6 represents the diffraction peak at 2*θ* values of 31.01°, 36.25°, 38.62°, and 42.2° corresponding to the crystal plane; (2 2 0), (3 1 1), (2 2 2), and (4 0 0) represent the crystalline structure of iron oxide nanoparticles.

### 3.7. Antimicrobial Activity

**Figure 7:**
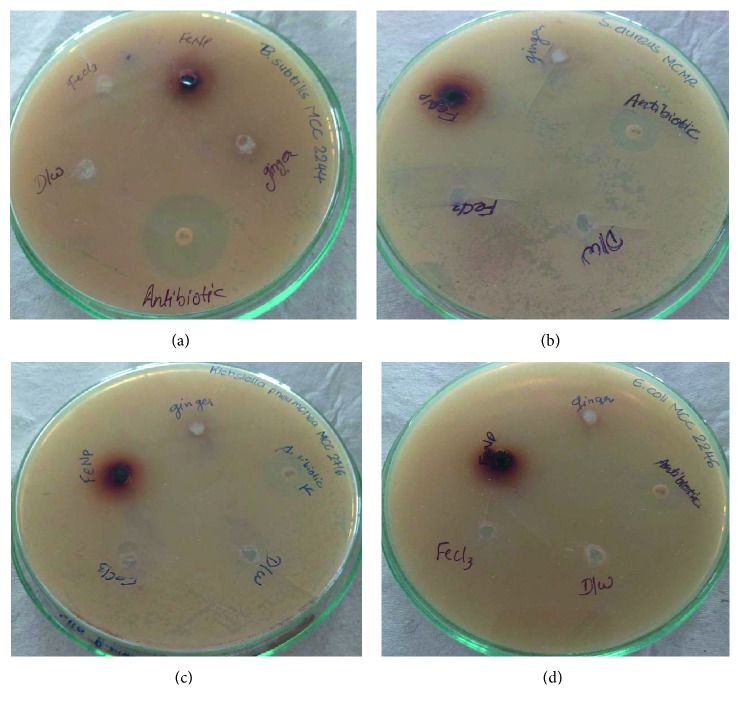
Antimicrobial activity of GR-FeNPs against (a) *Bacillus subtilis*, (b) *Staphylococcs aureus*, (c) *Klebsiella pneumoniae*, and (d) *Escherichia coli*.

Various Gram-positive and Gram-negative bacterial strains were used to check the bactericidal activity of FeNPs synthesized via green chemistry. The excessive use of antibiotics has led to the emergence of new multidrug-resistant strains. It is necessary to find an alternative to antibiotics. Earlier studies reported the use of expensive metal nanoparticles as antimicrobial agents [31–33]. To overcome this problem, the current study's focus was to design an eco-friendly and cost-effective method. The results are shown in Table 3. *E. coli* and *K. pneumoniae* show more sensitivity than Gram-positive bacteria. Compared with Gram-positive bacteria, Gram-negative bacteria have a thin layer of peptidoglycan. Hence, FeNPs can easily penetrate the cell wall of Gram-negative bacteria (Figure 7). Our results support the notion of earlier studies that *E. coli* and *K. pneumoniae* showed higher zones of inhibition than *B. subtilis* and *S. aureus* [26, 27, 34].

### 3.8. Antimicrobial Activity of Iron Nanoparticle-Coated Surgical Cotton

**Figure 8:**
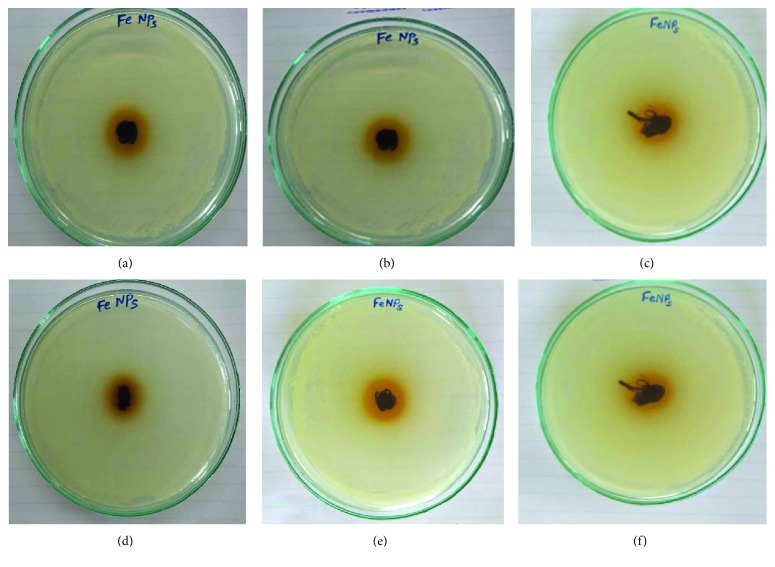
Antimicrobial activity of GR-FeNPs-coated surgical cotton after 24 hrs against (a) *Bacillus subtilis*, (b) *Staphylococcs aureus*, and (c) *Escherichia coli*, and after 30 days against (d) *Bacillus subtilis*, (e) *Staphylococcs aureus*, and (f) *Escherichia coli*.

The key outcome of the present study is the FeNPs-coated surgical cotton. The bactericidal activity of FeNPs extended to the surgical cotton, and it can be used further in wound healing, tissue therapy, and other medicinal applications. 10 *μ*g/ml of FeNPs was used to coat the surgical cotton with the help of a dip coater. The antimicrobial activity was evaluated on Gram-positive *B. subtilis*, *S. aureus*, and Gram-negative *E. coli*, by the standard disc diffusion method. The results are given in Table 4. The results showed the radial diameter of the inhibiting zones of *B. subtilis*, *S. aureus*, and *E. coli* after 24 hours. The clear inhibition zones made by the FeNPs-coated surgical cotton obtained in the present study are shown in Figure 8. Antimicrobial activity was evaluated on day zero and 30 days after coating. Initially, the particles showed higher antimicrobial activity, which diminished in terms of zone diameter due to the development of resistance in the microbial culture used for the study. The clear zone even after 30 days indicates bacterial growth restriction by the diffused FeNPs over the surgical cotton. Furthermore, the green approach for synthesis of FeNPs can be applied on cotton fabric which could have good bactericidal activity in wound dressing [30, 35–38].

### 3.9. Antioxidant Activity

**Figure 9:**
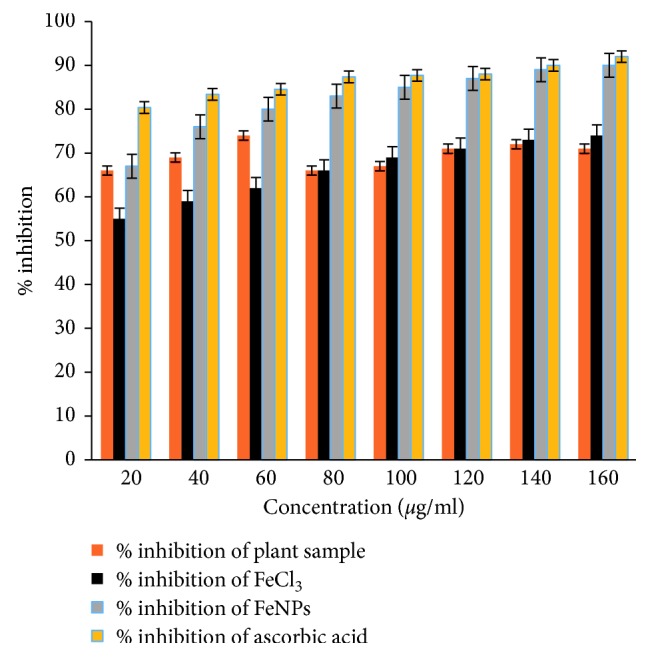
Antioxidant activity of GR-FeNPs.

*Z. officinale* is well known for its herbal properties as it is used in Chinese and Indian medicine since ancient times. The antioxidant property of ginger is due to the presence of phytochemicals such as gingerol, vitamin C, ß carotene, flavonoids, and tannins. Life on Earth without oxygen is impossible, and oxygen is also a well-known reactive molecule that causes damage to living organisms by producing reactive oxygen species. Our body is a complex network of antioxidant metabolites and enzymes that work together to prevent oxidative damage to cellular components. The main function of reactive oxygen species is signalling redox reactions. Thus, the function of an antioxidant system is not to remove oxidants entirely but instead to maintain an optimum level inside the body. Ascorbic acid has high amounts of antioxidants; thus, it was used as standard (Figure 9). By using the DPPH assay at different sets of concentrations, antimicrobial activity was evaluated in triplicate.

The total antioxidant capacity of *Z. officinale* was expressed as the number of equivalents of ascorbic acid. The color of the DPPH solution in the presence of the GR-FeNPs changes gradually from deep violet to pale yellow, which allowed the visual monitoring of the antioxidant activity of the nanoparticles. The observed effect of FeNPs is in the following order: ascorbic acid > FeNPs > FeCl_3_ > plant extract [Figure 8]. The study revealed that the antioxidant activity of the extract follows an increasing trend with the increase in concentration of the GR-FeNPs. DPPH activity results showed the highest free radical % scavenging potentials of 0.01 M FeCl_3_, GR extract, GR-Fe NPs, and ascorbic acid to be 74%, 71%, 89%, and 92%, respectively, at a concentration of 160 *μ*g/mL [39, 40].

## 4. Conclusion

The present work highlighted the green chemistry of synthesizing iron nanoparticles from the root extract of *Z. officinale*. The production proved to be easy, cost effective, and eco-friendly with natural reagents and less harsh chemicals. The color change was also remarkable when ferric chloride solution was mixed with the reducing agent of the plant extract. The biosynthesized FeNPs were characterized by UV-Vis spectroscopy that showed a surface plasmon resonance behaviour. The antimicrobial activity reported using green approach-synthesized nanoparticles may be further beneficial for various applications for better prognosis of several diseases, and the antioxidant activity of *Z. officinale* root extract has shown tremendous results. Iron nanocoated surgical cotton would have great role for distinguished health applications by creating new gadgets such as biosensors which can be implemented for the study may enhance the effects of conventional antimicrobials, which will probably decrease costs and improve the treatment quality. Thus, nanocoated surgical cotton obtained using a green synthesis approach might be a promising path in the field of medical microbiology. Future studies are still needed to design nanochips having an antimicrobial property which can replace antibiotics in our lives.

## Figures and Tables

**Table 1:** Phytochemical GC-MS analysis of *Zingiber officinale* root extract.

Peak	Compounds	Start	RT (min)	End	Area (%)
1	Propanol	2.196	2.213	2.459	6.25
2	2-Hexanol, 2-methyl	2.654	2.705	2.768	5.34
3	Benzene, 1,3-dimethyl(1S,5S)-2-methyl-5-((R)-6-methylhept-5-en-2-yl)bicyclo[3.1.0]hex-2-ene	2.768	2.797	2.865	1.34
4	(1S,5S)-2-methyl-5-((R)-6-methylhept-5-en-2-yl)bicyclo[3.1.0]hex-2-ene	17.062	17.176	17.279	11.84
5	Alpha.-farnesene	17.388	17.48	17.542	4.19
6	Beta.-bisabolene	17.731	17.806	17.889	4.11
7	Gingerol	20.529	20.735	20.81	18.15
8	Trans-sesquisabinene hydrate	33.281	33.541	33.65	27.63
9	Gingerol	34.222	34.411	34.611	8.79
10	3-Decanone, 1-(4-hydroxy-3-methoxyphenyl)	35.767	36.288	36.328	100
11	7-Oxabicyclo[4.1.0]heptane, 1-(2,3-dimethyl-1,3-butadienyl)-2,2,6-trimethyl-, (E)	37.055	37.14	37.249	6.34
12	Gingerol	37.455	37.53	37.627	7.83
13	1-(4-Hydroxy-3-methoxyphenyl)dec-4-en-3-one	37.747	37.804	37.839	5.26
14	Cedrol	37.839	37.879	37.947	4.22
15	Gingerol	37.947	37.982	38.01	2
16	Gingerol	38.01	38.045	38.176	2.97
17	1-(4-Hydroxy-3-methoxyphenyl)tetradec-4-en-3-one	38.302	38.411	38.625	18.28
18	Limonen-6-ol, pivalate	39.258	39.332	39.446	14
19	Oligandrol	39.612	39.687	39.795	10.59
20	Gingerol	40.328	40.368	40.419	3.06

**Table 2:** Comparison of bond stretching of synthesized FeNPs with *Z. officinale* root extract.

Samples	C-H bond stretching	C-C bond stretching	C-N bond stretching
*Zingiber officinale* root extract	2857 cm^−1^	1514 cm^−1^	1037 cm^−1^
FeNPs synthesized from *Zingiber officinale* root extract	2927 cm^−1^	1636 cm^−1^	1075 cm^−1^

**Table 3:** Diameter of the zone of inhibition of GR-FeNPs against Gram-positive and Gram-negative bacteria.

Pathogens	Diameter of the zone of inhibition (mm)				
	GR-FeNPs (30 *µ*g/ml)	Antibiotic (kanamycin 30 *µ*g/disk)	FeCl_3_ (0.01 M)	Plant extract	Distilled water
*Bacillus subtilis* MCC 2244	10 ± 0.2	20 ± 0.4	5 ± 0.3	3 ± 0.2	NO
*Escherichia coli* MCC 2246	18 ± 0.34	13 ± 0.4	8 ± 0.2	2 ± 0.1	NO
*Klebsiella pneumoniae* MCC 2716	16 ± 0.4	12 ± 0.3	7 ± 0.3	2 ± 0.2	NO
*Staphylococcs aureus* MCC 2408	13 ± 0.23	15 ± 0.3	5 ± 0.4	3 ± 0.1	NO

NO: not observed

**Table 4:** Antimicrobial activity of GR-FeNPs-coated surgical cotton.

Pathogens	Diameter of zone of inhibition (24 hours)	Diameter of zone of inhibition (30 days)
*Bacillus subtilis* (MCC 2244)	9 mm ± 0.24 mm	5 mm ± 0.4 mm
*Staphylococcus aureus* (MCC 2408)	12 mm ± 0.28 mm	11 mm ± 0.29 mm
*Escherichia coli* (MCC 2246)	14 mm ± 0.3 mm	6 mm ± 0.3 mm

## Data Availability

GC/MS analysis data is available in supplementary files, rest of the data are available within manuscript.

## Supplementary Materials

Supplementary Materials The supplementary material includes detailed GC/MS chromatograph and structure of phytochemicals present in the root extract of *Z. officinale*. Based upon the supplementary data, the retention time of each phytochemical has been included in the current study.

## References

[1] Prucek R., Tuček J., Kilianová M. (2011). The targeted antibacterial and antifungal properties of magnetic nanocomposite of iron oxide and silver nanoparticles. *Biomaterials*.

[2] Noguez C. (2007). Surface plasmons on metal nanoparticles: the influence of shape and physical environment. *Journal of Physical Chemistry C*.

[3] Zhang Y., Yang Y., Tang K., Hu X., Zou G. (2007). Physicochemical characterization and antioxidant activity of quercetin-loaded chitosan nanoparticles. *Journal of Applied Polymer Science*.

[4] Mittal A. K., Kaler A., C Banerjee U. (2012). Free radical scavenging and antioxidant activity of silver nanoparticles synthesized from flower extract of *Rhododendron dauricum*. *Nano Biomedicine and Engineering*.

[5] Wu Y., Luo Y., Wang Q. (2012). Antioxidant and antimicrobial properties of essential oils encapsulated in zein nanoparticles prepared by liquid-liquid dispersion method. *LWT–Food Science and Technology*.

[6] Mageswari A., Srinivasan R., Subramanian P., Ramesh N., Gothandam K. M. (2016). Nanomaterials: classification, biological synthesis and characterization. *Nanoscience in Food and Agriculture*.

[7] Liu X., Dai Q., Austin L. (2008). A one-step homogeneous immunoassay for cancer biomarker detection using gold nanoparticle probes coupled with dynamic light scattering. *Journal of the American Chemical Society*.

[8] Laurent S., Forge D., Port M. (2008). Magnetic iron oxide nanoparticles: synthesis, stabilization, vectorization, physicochemical characterizations, and biological applications. *Chemical reviews*.

[9] Dhalani J., Kapadiya K., Pandya M., Dubal G., Imbraj P., Nariya P. (2018). An approach to identify sterol entities from abrus precatorius’s seeds by GC-MS. *NISCAIR-CSIR*.

[10] Turakhia B., Turakhia P., Shah S. (2018). Green synthesis of zero valent iron nanoparticles from *Spinacia oleracea* (spinach) and its application in waste water treatment. *Journal for Advanced Research in Applied Sciences*.

[11] Turakhia B., Chapla K., Turakhia P. (2017). Green synthesis of zero valent iron nano particles from *Coriandrum sativum* (Coriander) and its application in reduction chemical oxygen demand and biological oxygen demand in waste water. *South-Asian Journal of Multidisciplinary Studies*.

[12] Shahwan T., Abu Sirriah S., Nairat M. (2011). Green synthesis of iron nanoparticles and their application as a Fenton-like catalyst for the degradation of aqueous cationic and anionic dyes. *Chemical Engineering Journal*.

[13] Harshiny M., Iswarya C. N., Matheswaran M. (2015). Biogenic synthesis of iron nanoparticles using *Amaranthus dubius* leaf extract as a reducing agent. *Powder Technology*.

[14] Liu X., Zhang P., Li X. (2009). Trends for nanotechnology development in China, Russia, and India. *Journal of Nanoparticle Research*.

[15] Kim D. K., Zhang Y., Voit W., Rao K. V., Muhammed M. (2001). Synthesis and characterization of surfactant-coated superparamagnetic monodispersed iron oxide nanoparticles. *Journal of Magnetism and Magnetic Materials*.

[16] Arakha M., Pal S., Samantarrai D. (2015). Antimicrobial activity of iron oxide nanoparticle upon modulation of nanoparticle-bacteria interface. *Scientific Reports*.

[17] Azam A., Ahmed A. S., Oves M., Khan M. S., Habib S. S., Memic A. (2012). Antimicrobial activity of metal oxide nanoparticles against Gram-positive and Gram-negative bacteria: a comparative study. *International Journal of Nanomedicine*.

[18] Mahdy S. A., Raheed Q. J., Kalaichelvan P. T. (2012). Antimicrobial activity of zero-valent iron nanoparticles. *International Journal of Modern Engineering Research*.

[19] Reneker D. H., Chun I. (1999). Nanometre diameter fibres of polymer, produced by electrospinning. *Nanotechnology*.

[20] Raghavendra G. M., Jayaramudu T., Varaprasad K., Sadiku R., Ray S. S., Mohana Raju K. (2013). Cellulose-polymer-Ag nanocomposite fibers for antibacterial fabrics/skin scaffolds. *Carbohydrate Polymers*.

[21] Machado S., Pinto S. L., Grosso J. P., Nouws H. P. A., Albergaria J. T., Delerue-Matos C. (2013). Green production of zero-valent iron nanoparticles using tree leaf extracts. *Science of the Total Environment*.

[22] Ševců A., El-Temsah Y. S., Joner E. J., Černík M. (2011). Oxidative stress induced in microorganisms by zero-valent iron nanoparticles. *Microbes and Environments*.

[23] Saikia J. P., Paul S., Konwar B. K., Samdarshi S. K. (2010). Ultrasonication: enhances the antioxidant activity of metal oxide nanoparticles. *Colloids and Surfaces B: Biointerfaces*.

[24] Rahman S. S. U., Qureshi M. T., Sultana K. (2017). Single step growth of iron oxide nanoparticles and their use as glucose biosensor. *Results in Physics*.

[25] Balamurughan M. G., Mohanraj S., Kodhaiyolii S., Pugalenthi V. (2014). Ocimum sanctum leaf extract mediated green synthesis of iron oxide nanoparticles: spectroscopic and microscopic studies. *Journal of Chemical and Pharmaceutical Sciences ISSN*.

[26] Jeyasundari J., Praba P. S., Jacob Y. B. A., Vasantha V. S., Shanmugaiah V. (2017). Green synthesis and characterization of zero valent iron nanoparticles from the leaf extract of *Psidium guajava* plant and their antibacterial activity. *Chemical Science Review and Letters*.

[27] Devatha C. P., Patil K. M. (2018). Effect of green synthesized iron nanoparticles by *Azardirachta indica* in different proportions on antibacterial activity. *Environmental Nanotechnology, Monitoring and Management*.

[28] Gottimukkala K. S. V., Harika R. P., Zamare D. (2017). Green synthesis of iron nanoparticles using green tea leaves extract. *Journal of Nanomedicine and Biotherapeutic Discovery*.

[29] Venkateswarlu S., Natesh Kumar B., Prasad C. H., Venkateswarlu P., Jyothi N. V. V. (2014). Bio-inspired green synthesis of Fe_3_O_4_ spherical magnetic nanoparticles using *Syzygium cumini* seed extract. *Physica B: Condensed Matter*.

[30] Phull A.-R., Abbas Q., Ali A. (2016). Antioxidant, cytotoxic and antimicrobial activities of green synthesized silver nanoparticles from crude extract of *Bergenia ciliata*. *Future Journal of Pharmaceutical Sciences*.

[31] Radulescu M., Andronescu E., Dolete G. (2016). Silver nanocoatings for reducing the exogenous microbial colonization of wound dressings. *Materials*.

[32] Prabhu S., Poulose E. K. (2012). Silver nanoparticles: mechanism of antimicrobial action, synthesis, medical applications, and toxicity effects. *International Nano Letters*.

[33] Zhou Y., Kong Y., Kundu S., Cirillo J. D., Liang H. (2012). Antibacterial activities of gold and silver nanoparticles against *Escherichia coli* and bacillus Calmette-Guérin. *Journal of Nanobiotechnology*.

[34] Devi A., Das V. K., Deka D. (2017). Ginger extract as a nature based robust additive and its influence on the oxidation stability of biodiesel synthesized from non-edible oil. *Fuel*.

[35] El-Shishtawy R. M., Asiri A. M., Abdelwahed N. A. M., Al-Otaibi M. M. (2010). In situ production of silver nanoparticle on cotton fabric and its antimicrobial evaluation. *Cellulose*.

[36] Lee H. J., Yeo S. Y., Jeong S. H. (2003). Antibacterial effect of nanosized silver colloidal solution on textile fabrics. *Journal of Materials Science*.

[37] Lee H. Y., Park H. K., Lee Y. M., Kim K., Park S. B. (2007). A practical procedure for producing silver nanocoated fabric and its antibacterial evaluation for biomedical applications. *Chemical Communications*.

[38] Tomšič B., Simončič B., Orel B. (2009). Antimicrobial activity of AgCl embedded in a silica matrix on cotton fabric. *Carbohydrate Polymers*.

[39] Abdel-Aziz M. S., Shaheen M. S., El-Nekeety A. A., Abdel-Wahhab M. A. (2014). Antioxidant and antibacterial activity of silver nanoparticles biosynthesized using *Chenopodium murale* leaf extract. *Journal of Saudi Chemical Society*.

[40] Bhattacharya K., Gogoi B., Buragohain A. K., Deb P. (2014). Fe_2_O_3_/C nanocomposites having distinctive antioxidant activity and hemolysis prevention efficiency. *Materials Science and Engineering: C*.

