# Supplementary material for: Novelty of Bioengineered Iron Nanoparticles in Nanocoated Surgical Cotton: A Green Chemistry
Source: Adv Pharmacol Sci. 2019 Feb 3;2019:9825969. doi: 10.1155/2019/9825969 (PMC6409075; doi:10.1155/2019/9825969)
Supplement: Supplementary Materials — The supplementary material includes detailed GC/MS chromatograph and structure of phytochemicals present in the root extract of Z. officinale. Based upon the supplementary data, the retention time of each phytochemical has been included in the current study. [file 9825969.f1.pdf]

RK University

Kasturbadham (Tramba), Rajkot-Bhavnagar Highway
Rajkot-360020

**Data Filename**	GF.D	**Sample Name**	GF
**Sample Type**		**Position**	7
**Instrument Name**	GCMS	**User Name**	
**Acq Method**	NATR JD .m	**Acquired Time**	11/29/2018 12:15:08 PM
**IRM Calibration Status**	Not Applicable	**DA Method**	RK University.m
**Comment**			

**Expected Barcode**		**Sample Amount**	
**Dual Inj Vol**	1	**TuneName**	ATUNE260Temp.U
**TunePath**	D:\MassHunter\GCMS\1\5977 \	**TuneDateStamp**	2018-10-09T09:43:46+05:30
**MSFirmwareVersion**	6.00.34	**OperatorName**	
**RunCompletedFlag**	True	**Acquisition SW Version**	MassHunter GC/MS Acquisition B.07.05.2479 23-Aug-2016 Copyright © 1989-2016 Agilent Technologies, Inc.

User Chromatograms

Fragmentor Voltage **Collision Energy** 0 **Ionization Mode** Unspecified

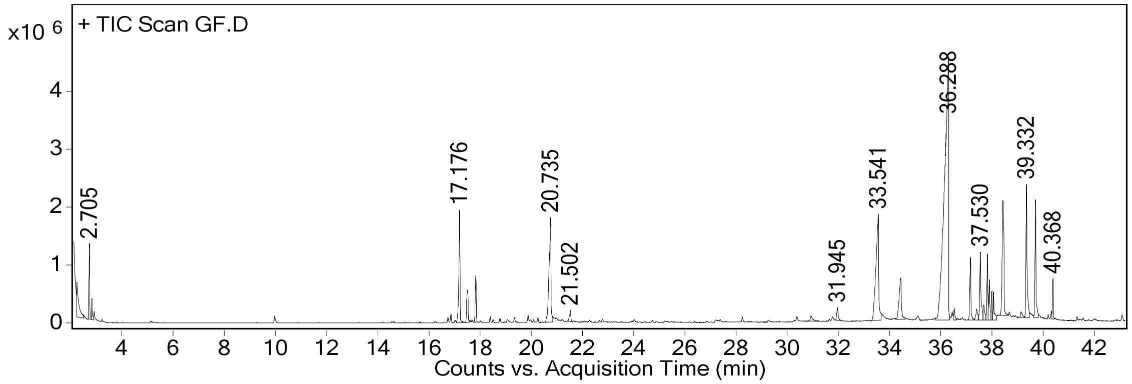

Integration Peak List

Peak	Start	RT	End	Height	Area	Area %	AreaSumPercent
1	2.196	2.213	2.459	599443.16	3180888.39	6.25	2.29
2	2.654	2.705	2.768	1306401.95	2715622.8	5.34	1.95
3	2.768	2.797	2.865	360621.32	679441.78	1.34	0.49
4	17.062	17.176	17.279	1949269.32	6023364.64	11.84	4.34
5	17.388	17.48	17.542	556293.71	2134465.81	4.19	1.54
6	17.731	17.806	17.889	805692.38	2093229.83	4.11	1.51
7	20.529	20.735	20.81	1809966.4	9236917.68	18.15	6.65
8	33.281	33.541	33.65	1838033.89	14062344.05	27.63	10.12
9	34.222	34.411	34.611	709896.89	4473573.69	8.79	3.22
10	35.767	36.288	36.328	4546606.47	50888312.02	100	36.63
11	37.055	37.14	37.249	1088004.5	3226818.66	6.34	2.32
12	37.455	37.53	37.627	1177329.5	3985259.96	7.83	2.87
13	37.747	37.804	37.839	1142581.5	2675587.1	5.26	1.93
14	37.839	37.879	37.947	698286.5	2149222.87	4.22	1.55

15	37.947	37.982	38.01	497434.5	1017992.32	2	0.73
16	38.01	38.045	38.176	484965.5	1510532.21	2.97	1.09
17	38.302	38.411	38.625	1989623.65	9302365.35	18.28	6.7
18	39.258	39.332	39.446	2307146.78	7123045.05	14	5.13
19	39.612	39.687	39.795	2037018.63	5388744.97	10.59	3.88
20	40.328	40.368	40.419	700603.58	1554967.83	3.06	1.12

User Spectra

Spectrum Source	Collision Energy	Ionization Mode
Peak (1) in "+ TIC Scan"	0	Unspecified

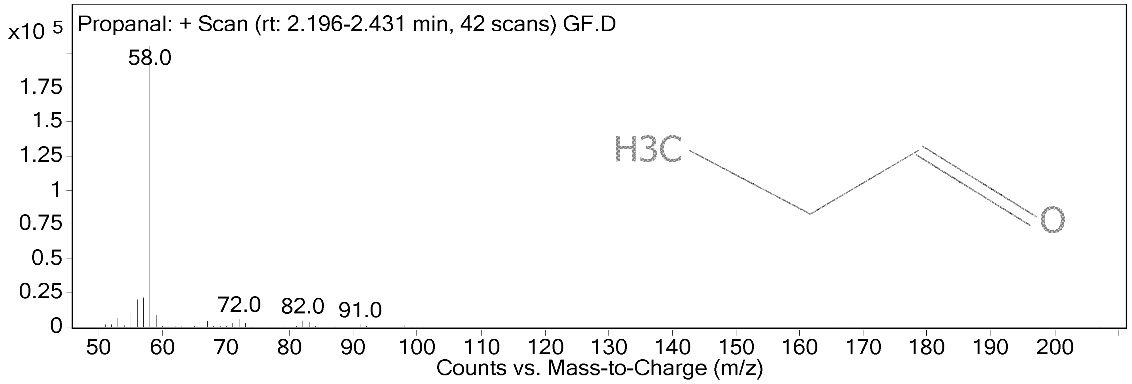

Peak List

m/z	Abund
51	1873.8
52	1754.51
53	6796.18
54	1571.45
55	11477.18
56.1	20156.13
57	21399.73
58	205364.36
59	8598.68
67	4113.5
71	2891.27
72	5749.13
73	2694.38
82	4613.6
83	3774
91	1960.12
98	1095.1

Spectrum Structure

Propanal

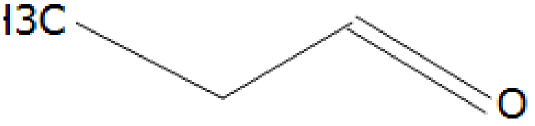

**Spectrum Source**	**Collision Energy**	**Ionization Mode**
Peak (2) in "+ TIC Scan"	0	Unspecified

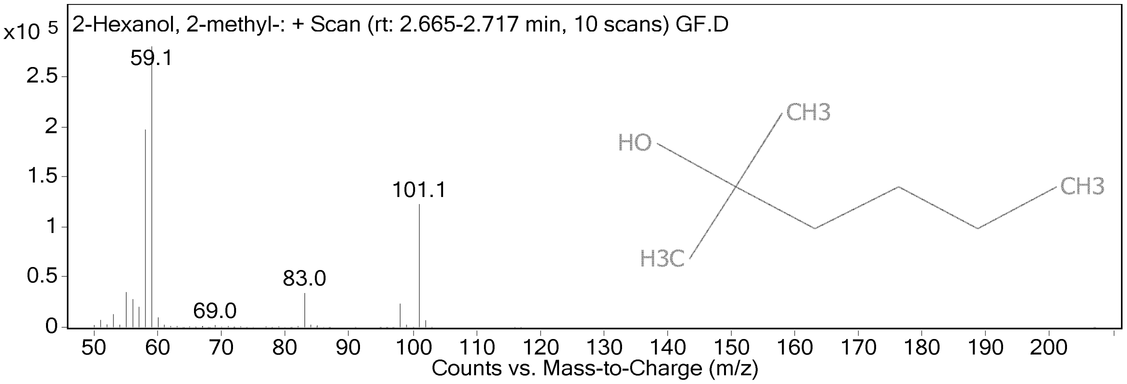

Peak List

m/z	Abund
50	2010.52
51	7102.7
52	2895.18
53	12968.96
54	2359.47
55.1	35003.39
56.1	27904.37
57	20240.1
58	197022.06
59.1	280250.22
60.1	9940.24
61	2580.42
69	2119.62
83	34207.13
84	2517.11
85	1902.12
98.1	23426.18
99.1	2609.2
101.1	122776.54
102.1	7064.19

Spectrum Structure
2-Hexanol, 2-methyl-

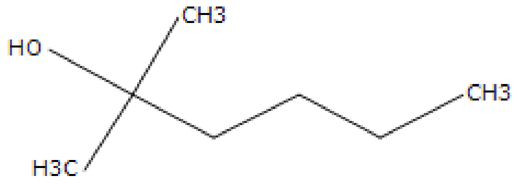

**Spectrum Source**	**Collision Energy**	**Ionization Mode**
Peak (3) in "+ TIC Scan"	0	Unspecified

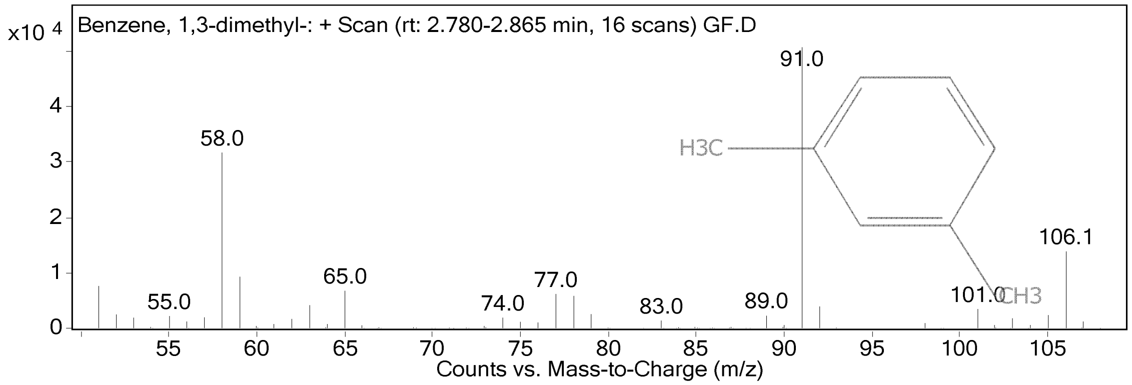

Peak List

m/z	**Abund**
51	7629.13
52	2499.03
53	1920.54
55	2161.11
57	1965.4
58	31752.77
59	9323.3
63	4153.72
65	6729.11
74	1894.87
77	6158.25
78	5819.77
79	2511.14
89	2264.31
91	50794.79
92	3911.24
101	3434.18
103	1749.53
105	2344.81

106.1	13840.39

Spectrum Structure

Benzene, 1,3-dimethyl-

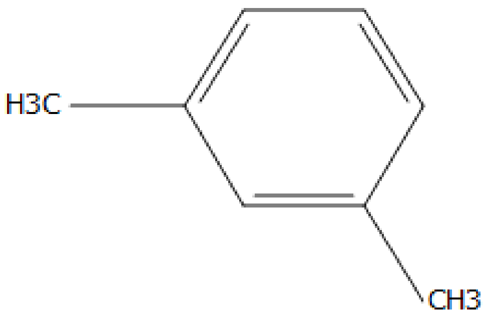

Spectrum Source
Peak (4) in "+ TIC Scan"

Collision Energy
0

Ionization Mode
Unspecified

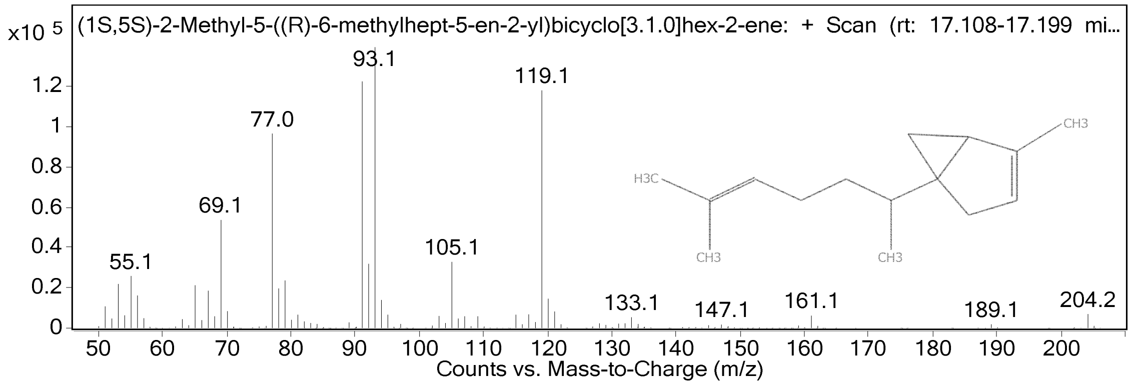

Peak List

m/z	Abund
51	10738.79
53.1	21810.92
55.1	25811.81
56.1	16232.44
65	21187.89
67.1	18538.32
69.1	53695.21
70.1	8450.98
77	96521.11
78	19692.9
79.1	23646.19
91.1	122411.41
92.1	31946.47
93.1	139479.86
94.1	13988.16
105.1	32878.41
119.1	118042.91

120.1	14593.96
121.1	8279.59
204.2	6874.86

Spectrum Structure

(1S,5S)-2-Methyl-5-((R)-6-methylhept-5-en-2-yl)bicyclo[3.1.0]hex-2-ene

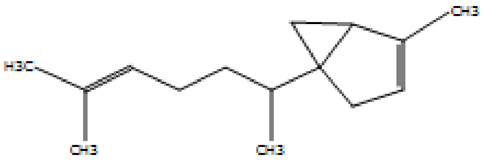

**Spectrum Source**	**Collision Energy**	**Ionization Mode**
Peak (5) in "+ TIC Scan"	0	Unspecified

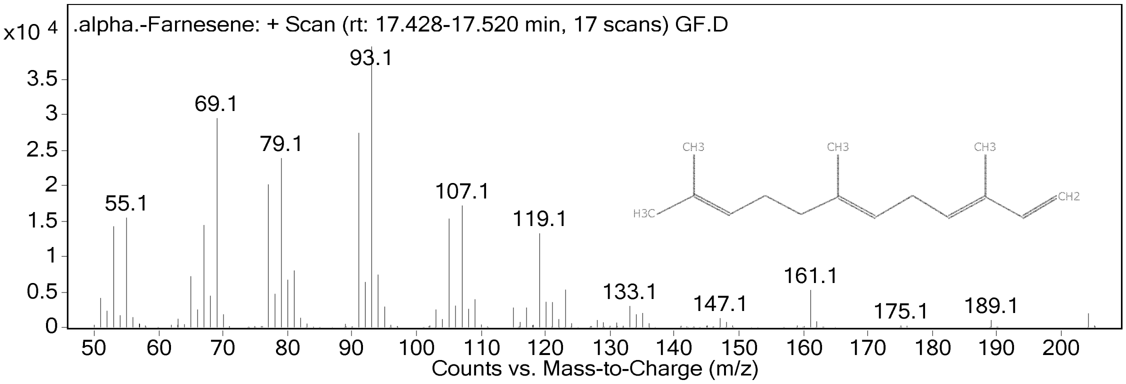

Peak List

m/z	Abund
53.1	14280.4
55.1	15496.41
65	7240.99
67.1	14469.45
68.1	4485.1
69.1	29558.06
77	20199.33
78	4716.5
79.1	23895.76
80	6729.9
81.1	8039.96
91.1	27469.82
92.1	6424.46
93.1	39712.16
94.1	7428.66

105.1	15345.72
107.1	17217.79
119.1	13276.5
123.1	5311.53
161.1	5262.13

Spectrum Structure

.alpha.-Farnesene

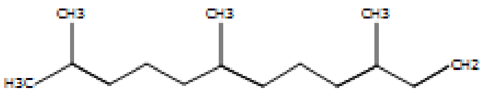

Spectrum Source
Peak (6) in "+ TIC Scan"

Collision Energy
0

Ionization Mode
Unspecified

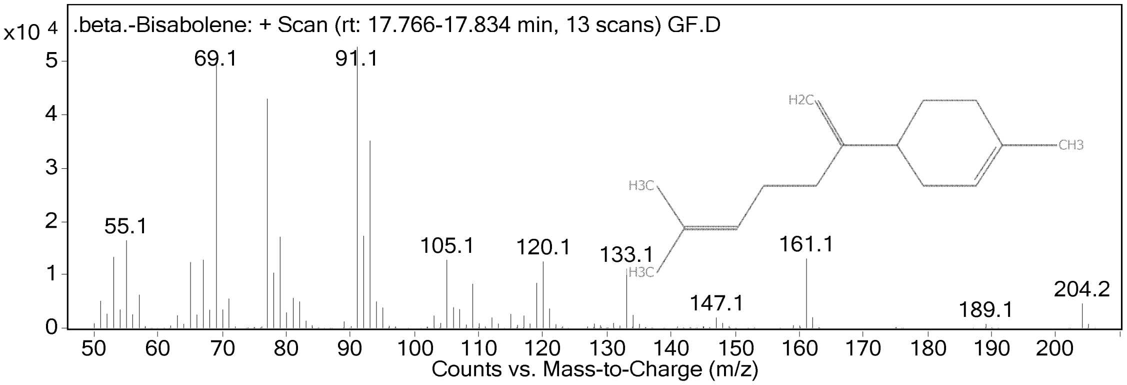

Peak List

m/z	Abund
53.1	13332.63
55.1	16454.47
57.1	6234.97
65	12378.8
67.1	12814.96
69.1	49427.14
71.1	5502.23
77	43088.92
78	10385.97
79.1	17109.12
81.1	5643.6
91.1	52841.94
92.1	17325.79

93.1	35189.57
105.1	12798.75
109.1	8297.74
119.1	8497.51
120.1	12480.75
133.1	11109.58
161.1	13054.55

Spectrum Structure

.beta.-Bisabolene

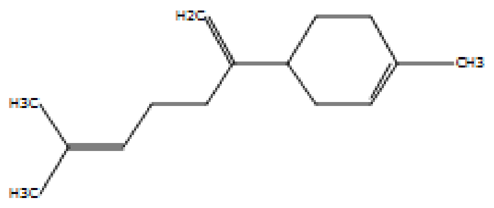

Spectrum Source	Collision Energy	Ionization Mode
Peak (7) in "+ TIC Scan"	0	Unspecified

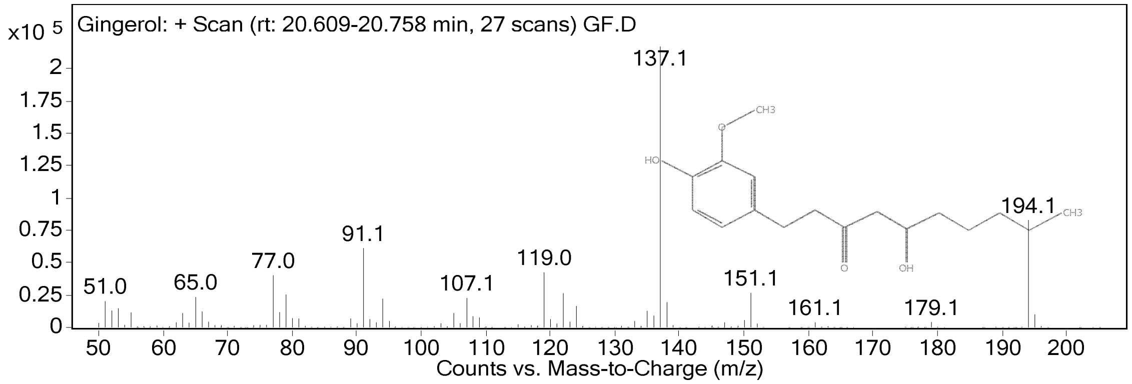

Peak List

m/z	Abund
51	20326.94
52	13093.04
53	14699.03
55	11490.08
65	23556.53
66.1	12194.12
77	40392.12
78.1	11730.62
79.1	25491.89
91.1	61266.13
94	22260.75

107.1	22770.26
119	42618.67
122	26302.12
124	16427.93
135	12681.99
137.1	217360.3
138.1	19540.85
151.1	26734.62
194.1	83120.66

Spectrum Structure

Gingerol

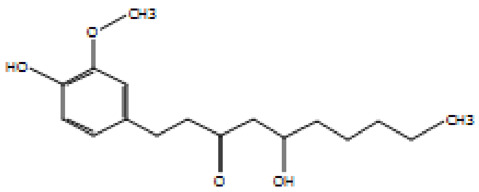

Spectrum Source Peak (8) in "+ TIC Scan"

Collision Energy 0

Ionization Mode Unspecified

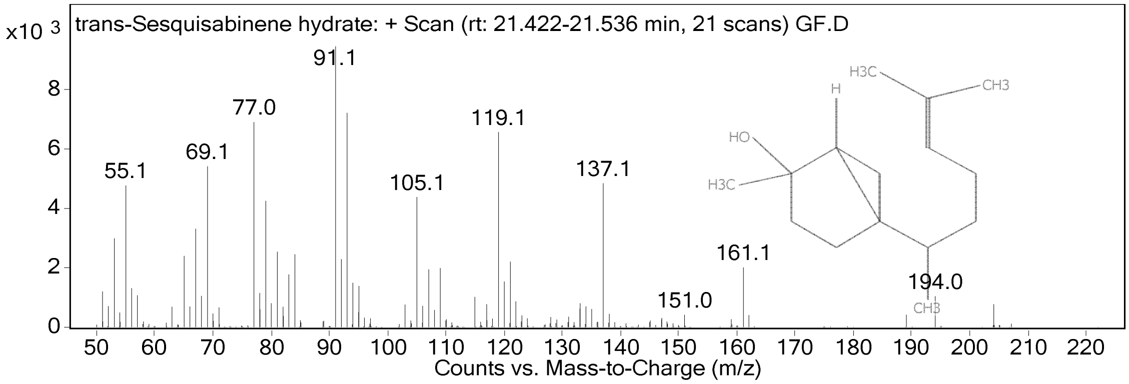

Peak List

m/z	Abund
53	2986.96
55.1	4768.65
65	2397.01
67	3317.65
69.1	5408.4
77	6901.75
79.1	4254.24
81.1	2532.78
83	1773.01

84.1	2451.9
91.1	9452.47
92	2289.84
93.1	7208.73
105.1	4378.92
107.1	1943.41
109.1	1985.06
119.1	6561.15
121.1	2200.19
137.1	4843.34
161.1	2013.8

Spectrum Structure

trans-Sesquisabinene hydrate

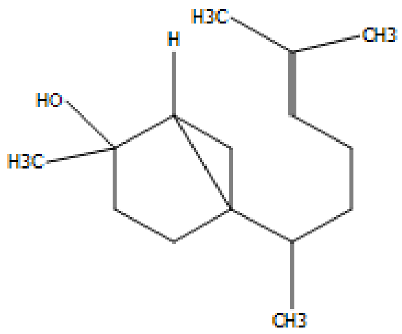

**Spectrum Source**	**Collision Energy**	**Ionization Mode**
Peak (9) in "+ TIC Scan"	0	Unspecified

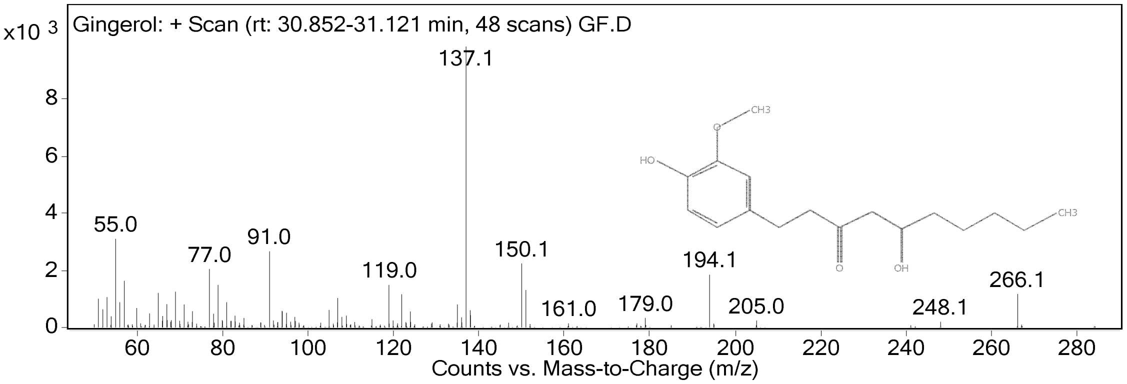

Peak List

m/z	**Abund**
51	1002.86
53	1056.35
55	3105.38
56	876.15
57.1	1636.23
65	1210.37
67	809.79

69	1254.67
77	2044.33
79	1481.16
81	883.69
91	2668
107	1030.72
119	1491.31
122	1163.04
137.1	9836.88
150.1	2234.11
151	1311.32
194.1	1847.42
266.1	1172.19

Spectrum Structure

Gingerol

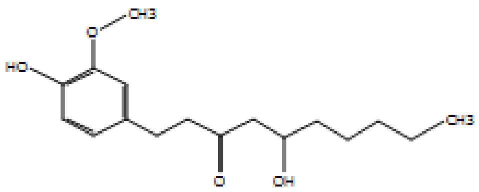

**Spectrum Source**	**Collision Energy**	**Ionization Mode**
Peak (10) in "+ TIC Scan"	0	Unspecified

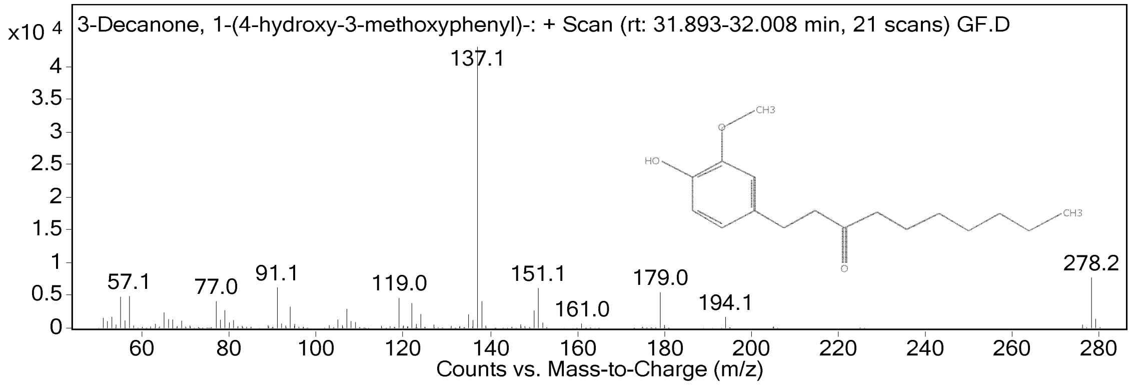

Peak List

m/z	Abund
53	1682.7
55.1	4808.01
57.1	4885.63
65	2391.03
77	4078.47
79	2731.53
91.1	6202.04
94	3288.03
107	2914.68
119	4609.3
122	3805.43
124	2127.29
135	2069.04
137.1	42998.01
138.1	4123.07
150.1	2672.22
151.1	6104.37
179	5483.18
194.1	1703.99

278.2	7715.96

Spectrum Structure

3-Decanone, 1-(4-hydroxy-3-methoxyphenyl)-

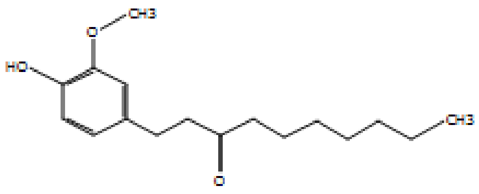

Spectrum Source Peak (11) in "+ TIC Scan"

Collision Energy 0

Ionization Mode Unspecified

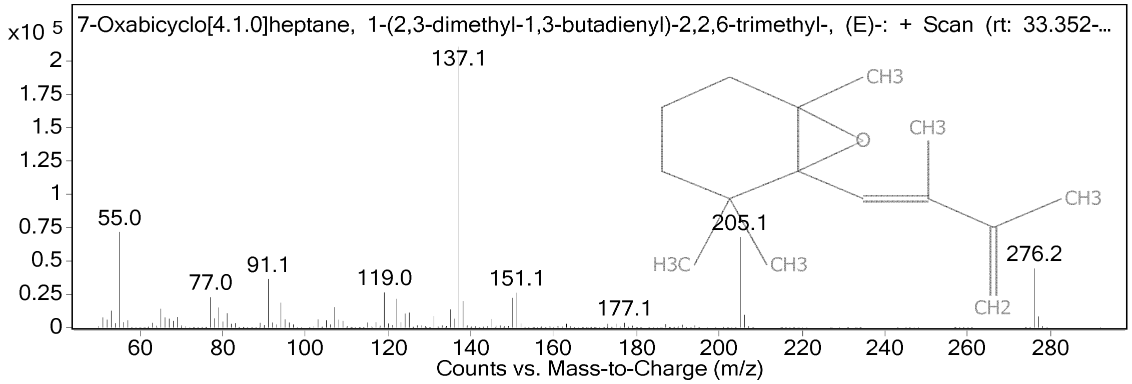

Peak List

m/z	Abund
53.1	12715.23
55	71790.09
65	14138.64
77	22895.23
79.1	15166.23
81	10699.34
91.1	36422.49
94	18763.16
107	15422.13
119	26475.35
122	21488.24
124	10565.06
125.1	11194.94
135	13743.06
137.1	211279.59
138.1	19939.51
150.1	22211.09

151.1	26201.68
205.1	68000.91
276.2	44534.48

Spectrum Structure

7-Oxabicyclo[4.1.0]heptane, 1-(2,3-dimethyl-1,3-butadienyl)-2,2,6-trimethyl-, (E)-

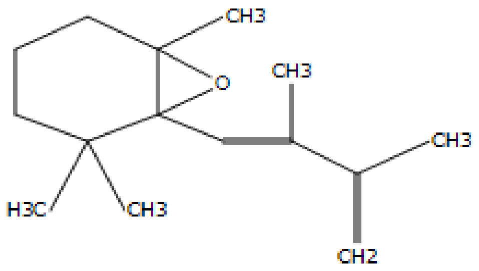

Spectrum Source	Collision Energy	Ionization Mode
Peak (12) in "+ TIC Scan"	0	Unspecified

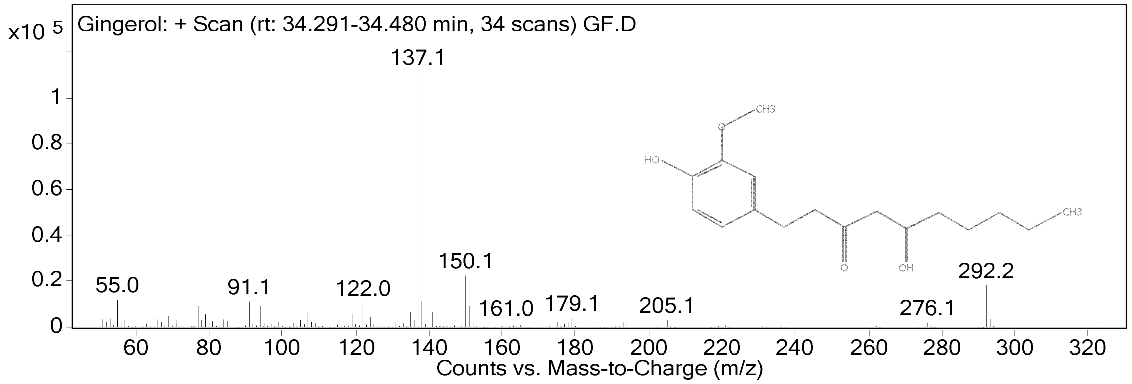

Peak List

m/z	Abund
53	3781.34
55	11875.6
65	5284.31
69	4843.67
77	9235.27
79.1	5533.95
91.1	11074.58
94	9158.48
107	6650.64
119	5855.52
122	10493.07
124	4429.33
135	6545.84
137.1	122585.83
138.1	11438.65

141.1	6631.44
150.1	22436.51
151.1	9299.04
179.1	3975.15
292.2	18200.85

Spectrum Structure

Gingerol

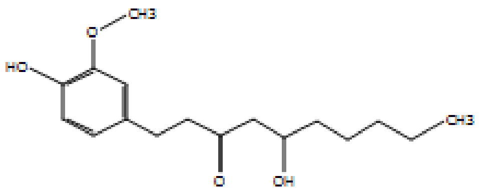

Spectrum Source Peak (13) in "+ TIC Scan" | **Collision Energy** 0 | **Ionization Mode** Unspecified

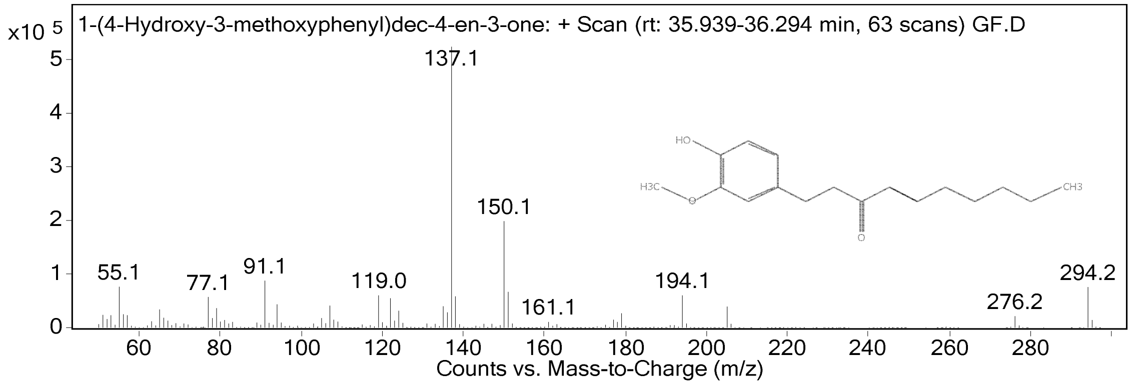

Peak List

m/z	Abund
55.1	76510.21
65.1	33789.27
77.1	56986.2
79.1	36597.36
91.1	87515.56
94	43685.46
107.1	40956
119	60058.34
122	54927.9
124	31312.19
135	39871.57
136.1	28759.7
137.1	525541.5

138.1	58644.54
150.1	199028.39
151.1	67027.5
179.1	26749.19
194.1	60067.98
205.1	39088.36
294.2	75769.72

Spectrum Structure

1-(4-Hydroxy-3-methoxyphenyl)dec-4-en-3-one

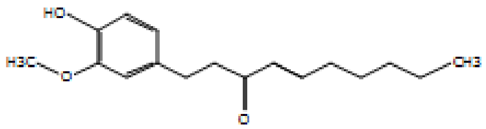

Spectrum Source
Peak (14) in "+ TIC Scan"

Collision Energy
0

Ionization Mode
Unspecified

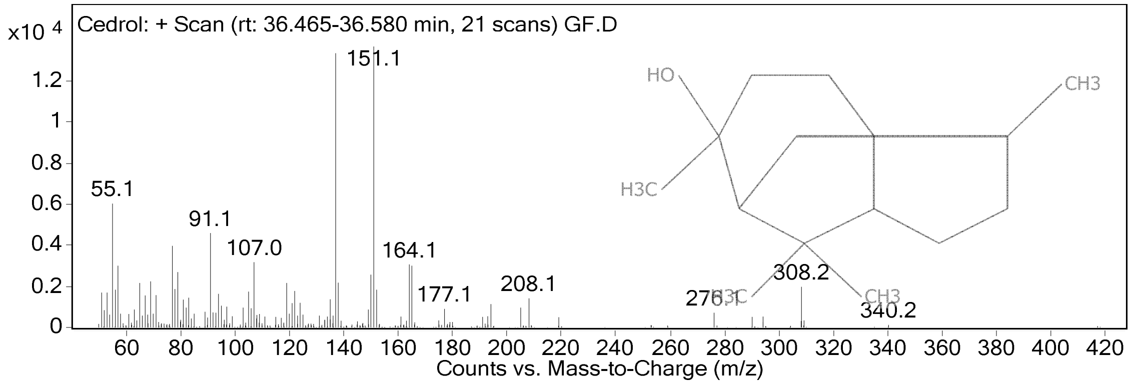

Peak List

m/z	Abund
55.1	6043.02
56.1	1830.96
57.1	3011.46
65	2162.55
69	2239.96
77	3968.82
78	1866.14
79	2694.32
91.1	4592.36
107	3176.06
119	2160.94

122	1777.3
137.1	13373.54
138	2181.71
150	2571.2
151.1	13703.24
152.1	1830.56
164.1	3062.5
165.1	3005.74
308.2	1978.01

Spectrum Structure

Cedrol

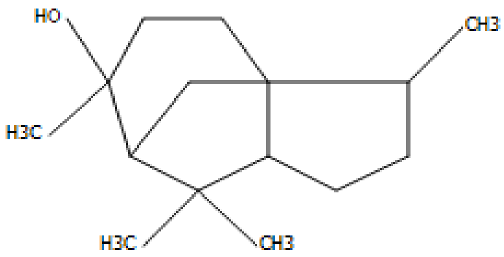

Spectrum Source
Peak (15) in "+ TIC Scan"

Collision Energy
0

Ionization Mode
Unspecified

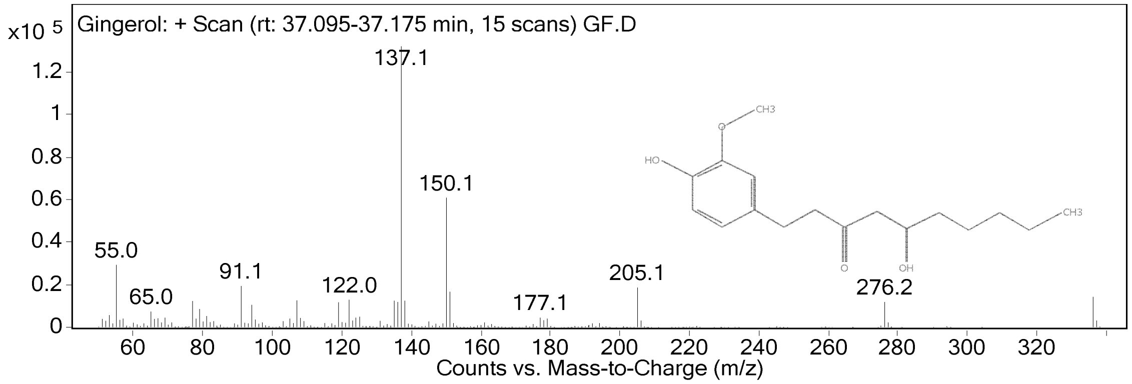

Peak List

m/z	**Abund**
53	5681.74
55	29411.13
65	7372.5
77	12387.93
79.1	8489.86
81.1	5248.72
91.1	19443.71
94	10484.6
107	12663.13

119	11723.73
122	12931.13
135	12482.93
136.1	11826.39
137.1	132343.39
138.1	12537.91
150.1	60947.15
151.1	16630.92
205.1	18669.99
276.2	11895.29
336.2	14285.9

Spectrum Structure

Gingerol

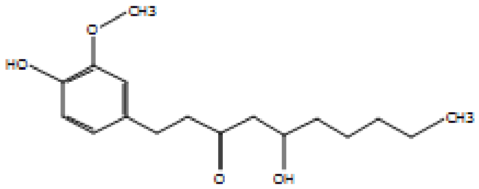

Spectrum Source
Peak (16) in "+ TIC Scan"

Collision Energy
0

Ionization Mode
Unspecified

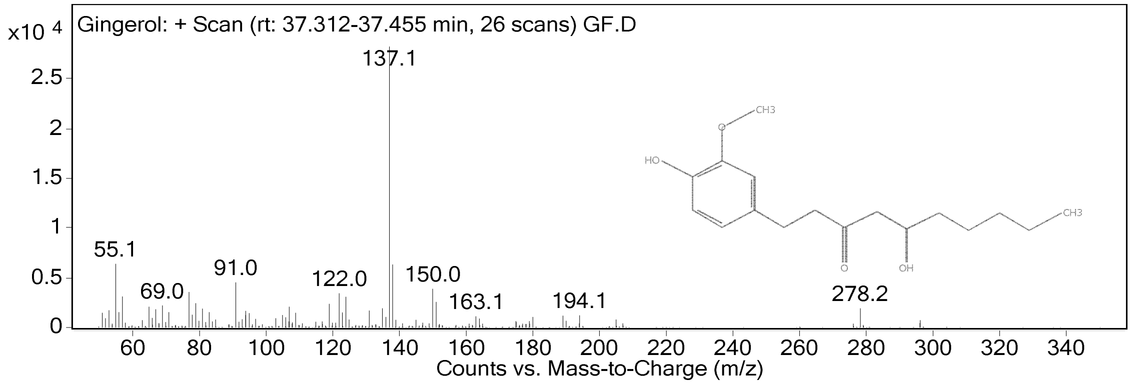

Peak List

m/z	Abund
53	1724.88
55.1	6394.03
57.1	3121.57
65	2087.58
67	1808.35
69	2208.98
77	3546.83

79	2432.98
81	1873.85
91	4532.68
107	2096.31
119	2362.02
122	3441.57
124	3082.66
135	1927.57
137.1	28231.69
138.1	6329.38
150	3867.84
151.1	2561
278.2	1904.96

Spectrum Structure

Gingerol

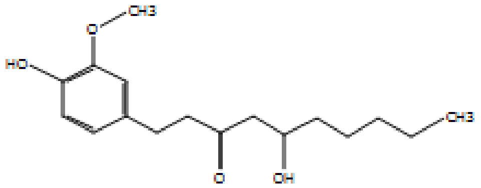

Spectrum Source Peak (17) in "+ TIC Scan"

Collision Energy 0

Ionization Mode Unspecified

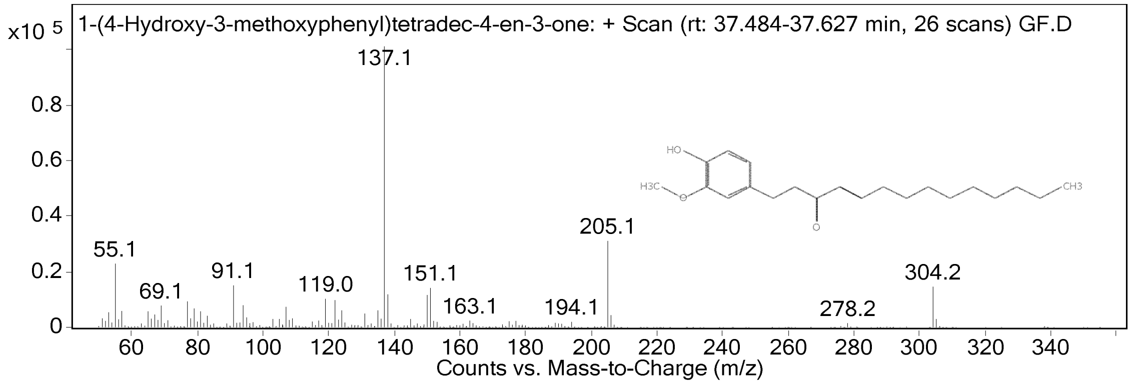

Peak List

m/z	Abund
55.1	22917.2
57.1	5831.63
65	5722.43
69.1	7772.82
77	9313.29

79.1	6753.53
81	5736.12
91.1	15032.13
94	7898.32
107	7301.1
119	10198.1
122	9773.86
124	6033.21
135	6053.53
137.1	101216.41
138.1	11820.45
150.1	11609.94
151.1	14191.67
205.1	31075.57
304.2	14621.83

Spectrum Structure

1-(4-Hydroxy-3-methoxyphenyl)tetradec-4-en-3-one

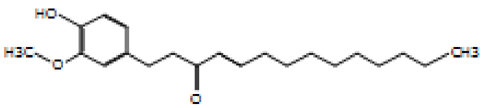

Spectrum Source Peak (18) in "+ TIC Scan"

Collision Energy 0

Ionization Mode Unspecified

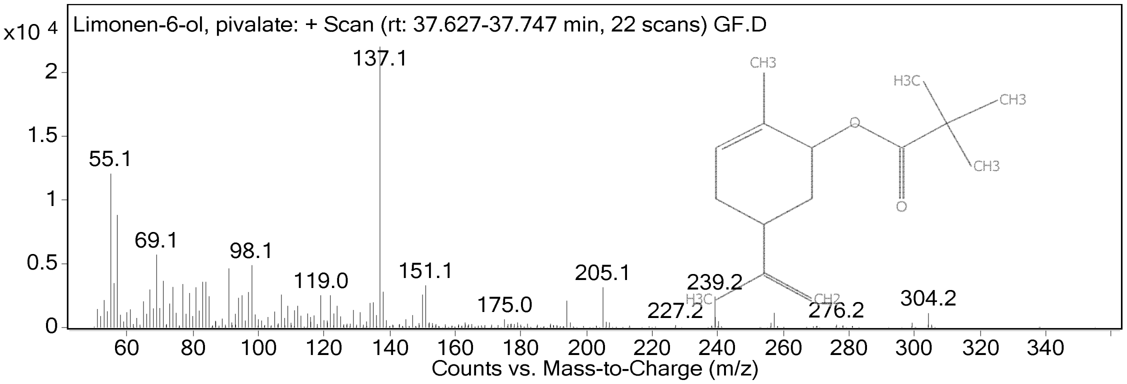

Peak List

m/z	Abund
55.1	12050.96
56.1	3487.13
57.1	8817.39

67	2975.52
69.1	5694.93
71.1	3644.82
74	3185.15
77	3391.44
79	2705.11
81.1	3156.35
83.1	3566.07
84.1	3583
91.1	4621.8
97.1	2775.7
98.1	4866.01
107	2583.97
137.1	21997.63
138	2801.73
151.1	3306.19
205.1	3156.48

Spectrum Structure

Limonen-6-ol, pivalate

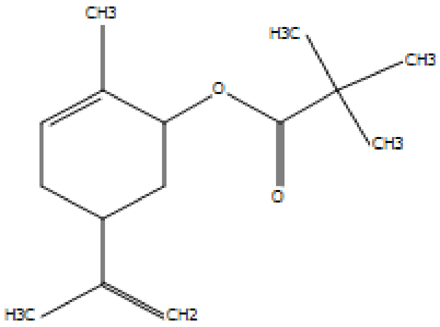

Spectrum Source	Collision Energy	Ionization Mode
Peak (19) in "+ TIC Scan"	0	Unspecified

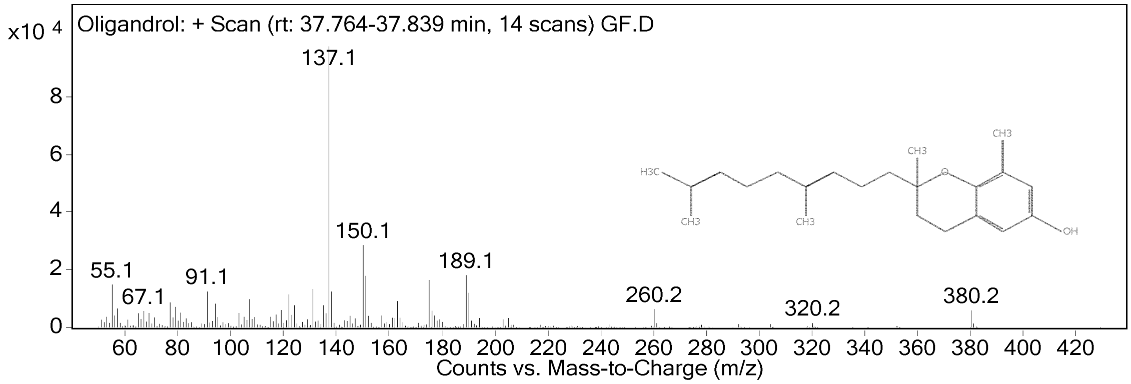

Peak List

m/z	Abund
55.1	14797.31

57.1	6560.32
77	8615.38
79.1	7116.26
91.1	12446.17
94	8211.51
107	9708.52
122	11366.97
124	7574.31
131	13244.68

m/z	Abund
135	7619.41
137.1	97512.88
138.1	12431.92
150.1	28495.67
151.1	17859.67
163.1	9115.01
175.1	16453.76
189.1	18052.49
190.1	11972.63
260.2	6268.48

Spectrum Structure

Oligandrol

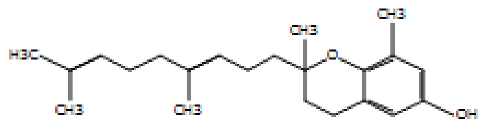

Spectrum Source
Peak (20) in "+ TIC Scan"

Collision Energy
0

Ionization Mode
Unspecified

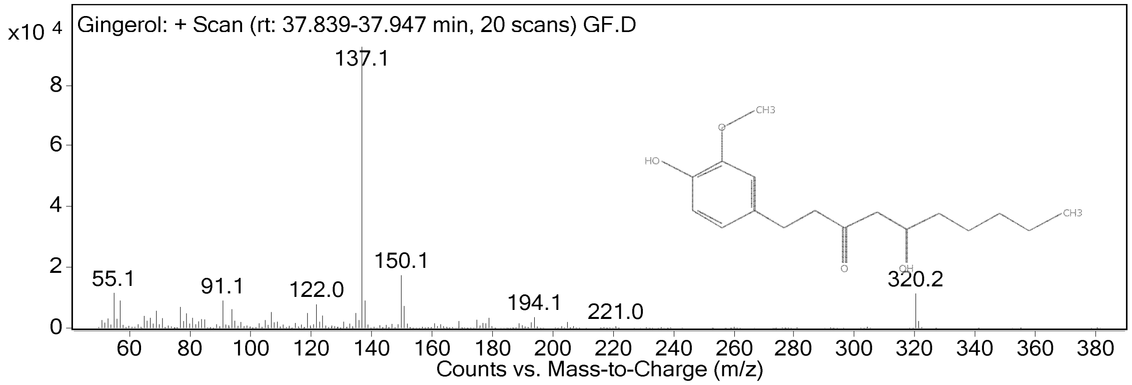

Peak List

m/z	Abund
55.1	11587.49
57.1	9037.34
65	3981.52
69.1	5638.84
77	6891.97
79.1	4806.83
81.1	3377.9
91.1	9033.3

94	6182.91
107	5261.88
119	4919.18
122	7803.96
124	4082.46
135	4880.25
137.1	92734.2
138.1	9039.38
150.1	17434.79
151.1	7290.77
194.1	3582.07
320.2	11350.12

Spectrum Structure

Gingerol

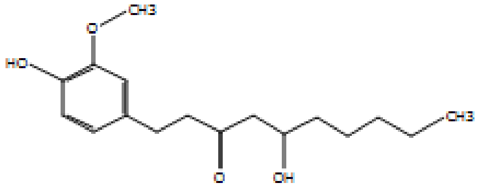

Spectrum Source Peak (21) in "+ TIC Scan"

Collision Energy 0

Ionization Mode Unspecified

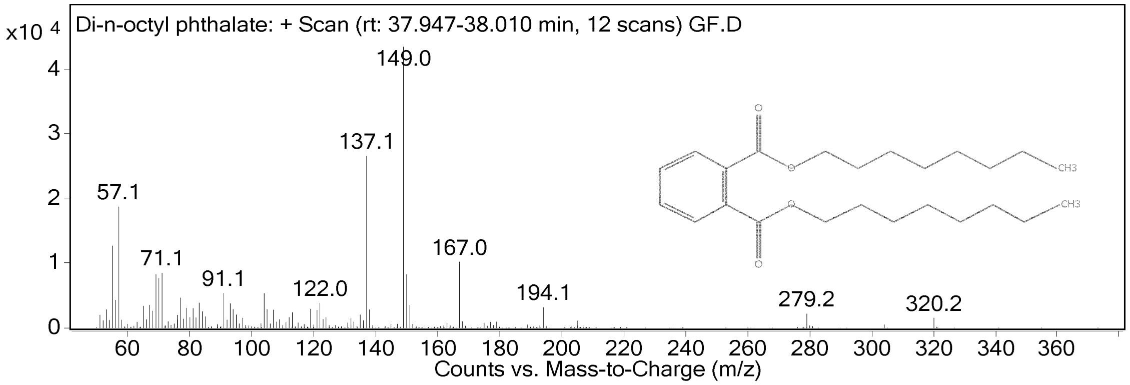

Peak List

m/z	**Abund**
55.1	12756.9
56.1	4374.52
57.1	18802.97
65	3427.62
67.1	3546.14
69.1	8310.72

70.1	7707.68
71.1	8535.69
77	4710.06
83.1	3929.68
91.1	5371.14
93	3816.79
104	5370.36
122	3826.04
137.1	26632.88
149	43575.43
150	8308
151.1	3574.34
167	10250.92
194.1	3233.74

Spectrum Structure

Di-n-octyl phthalate

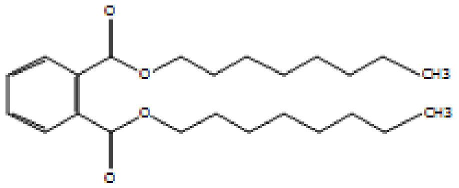

Spectrum Source
Peak (22) in "+ TIC Scan"

Collision Energy
0

Ionization Mode
Unspecified

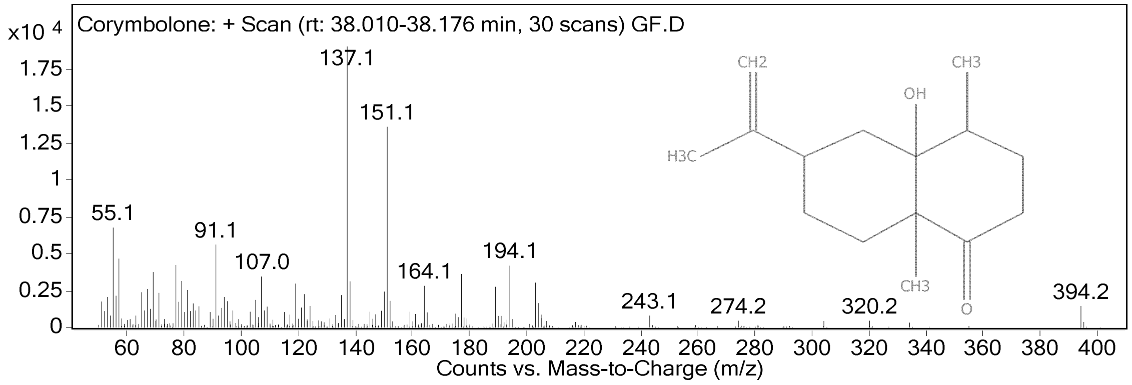

Peak List

m/z	Abund
55.1	6760.52
57.1	4645.27
65	2371.75
67	2584.32

69.1	3748.98
77	4211.66
79	3142.28
81	2518.94
91.1	5603.92
107	3446.64
119	2958.41
137.1	19022.96
138.1	3118
150.1	2416.04
151.1	13577.15
164.1	2801.08
177.1	3619.62
189.1	2745.24
194.1	4178.98
203.1	3026.51

Spectrum Structure

Corymbolone

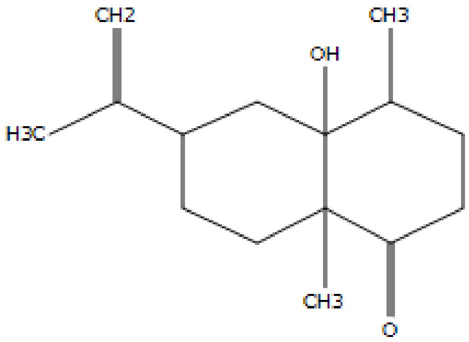

Spectrum Source Peak (23) in "+ TIC Scan"

Collision Energy 0

Ionization Mode Unspecified

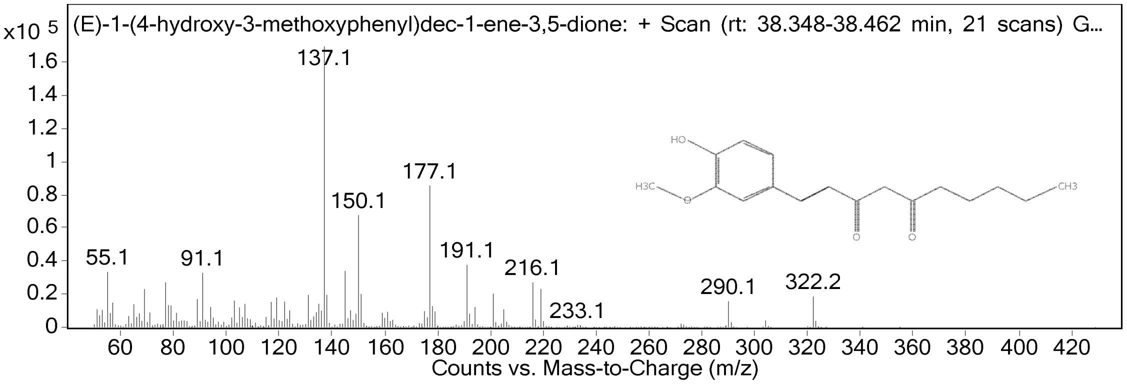

Peak List

m/z	Abund
55.1	33329.36
69.1	22947.5

77	26960.63
89	16956.87
91.1	32886.66
103.1	15939.19
119	18019.57
131	19493.65
137.1	169836.83
138.1	19382.99
145	34041.42
150.1	67593.06
151.1	19986.08
177.1	85553.48
191.1	37604.27
201.1	20177.66
216.1	27110.71
219.1	23163.76
290.1	15540.82
322.2	18600.16

Spectrum Structure

(E)-1-(4-hydroxy-3-methoxyphenyl)dec-1-ene-3,5-dione

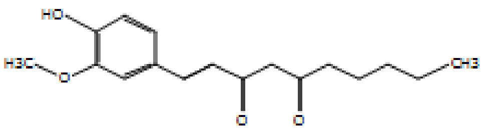

**Spectrum Source**	**Collision Energy**	**Ionization Mode**
Peak (24) in "+ TIC Scan"	0	Unspecified

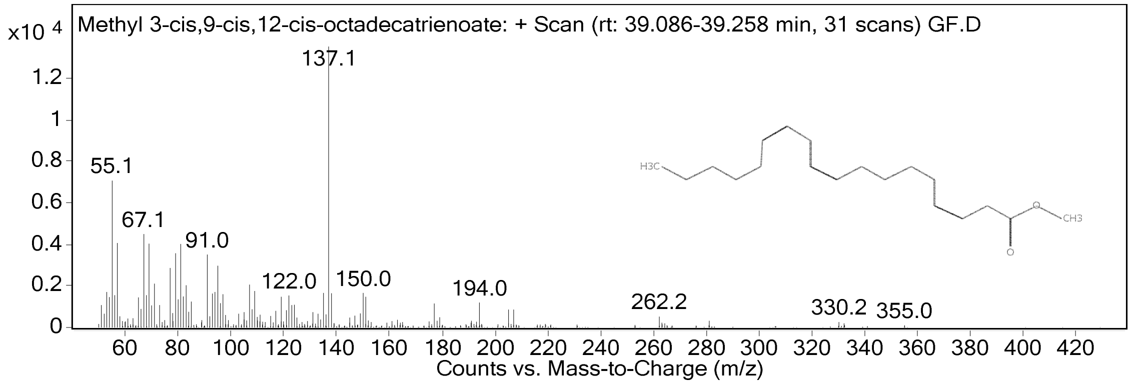

Peak List

m/z	**Abund**

53	1711.09
55.1	7085.52
57.1	4065.6
67.1	4502.9
69.1	4034.29
71.1	2102.6
77	2868.05
79	3570.16
81.1	4021.43
83.1	2034.84
91	3513.86
93.1	1633.52
94	1703.08
95.1	2974.37
107	2070.51
109.1	1759.28
135	1668.69
137.1	13550.34
138.1	1640.65
150	1656.53

Spectrum Structure

Methyl 3-cis,9-cis,12-cis-octadecatrienoate

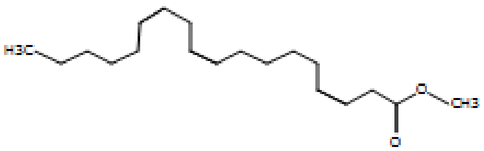

**Spectrum Source**	**Collision Energy**	**Ionization Mode**
Peak (25) in "+ TIC Scan"	0	Unspecified

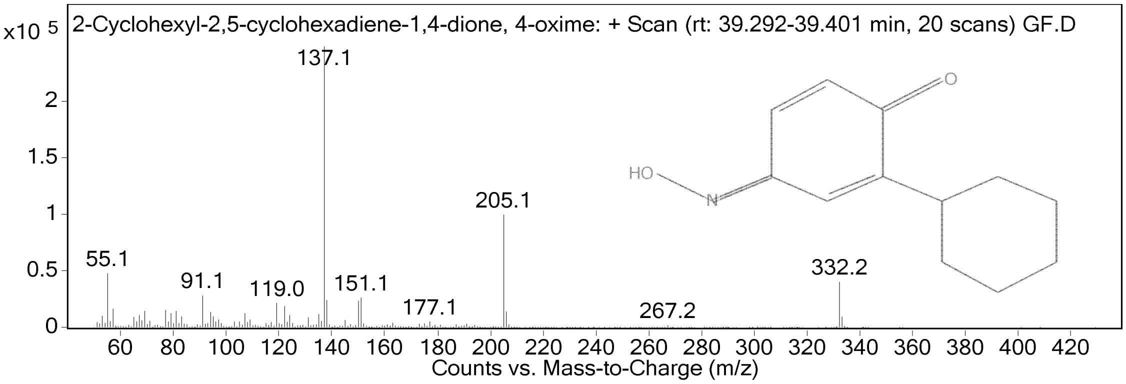

Peak List

m/z	Abund
55.1	47644.29
57.1	16245.88
69.1	14428.09
77	15205.11
79.1	12216.05
81.1	14342.58
91.1	28034.62
94	13544.09
107.1	12467.21
119	21445.84
122	18685.05
124	10556.71
135	11534.53
137.1	248293.25
138.1	23984.25
150.1	23360.88
151.1	26098.22
205.1	99437.08
206.1	13851.49
332.2	40090.12

Spectrum Structure

2-Cyclohexyl-2,5-cyclohexadiene-1,4-dione, 4-oxime

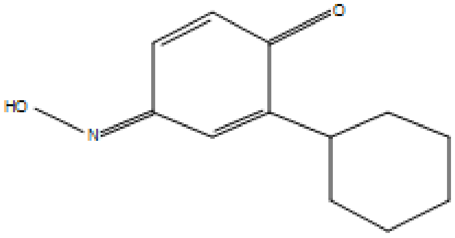

Spectrum Source	Collision Energy	Ionization Mode
Peak (26) in "+ TIC Scan"	0	Unspecified

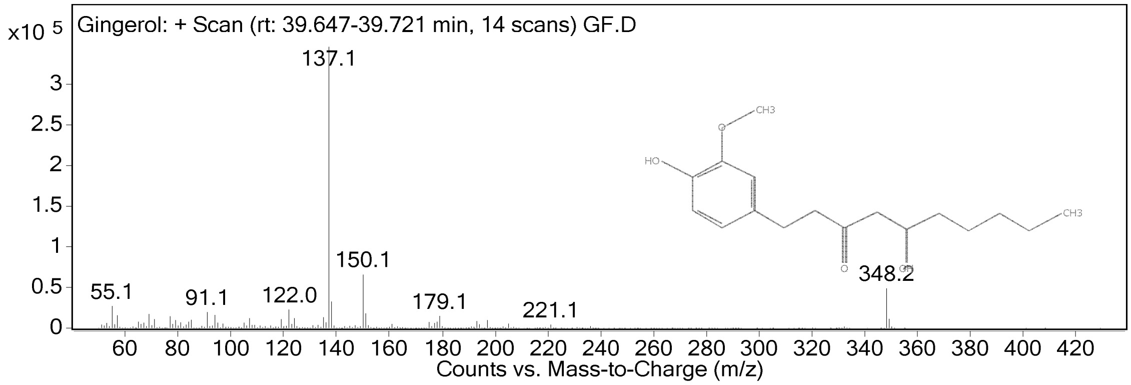

Peak List

m/z	Abund
55.1	26879.92
57.1	15573.95
69.1	17029.21
71.1	10712.39
77	14469.13
85.1	9921.49
91.1	19363.94
94	15972.29
107.1	11995.13
119	10771.74
122	22688.46
124	11961.46
135	13434.47
137.1	345522.19
138.1	32482.65
150.1	65747.96
151.1	17993.72
179.1	14714.78
348.2	48774
349.2	11439.71

Spectrum Structure

Gingerol

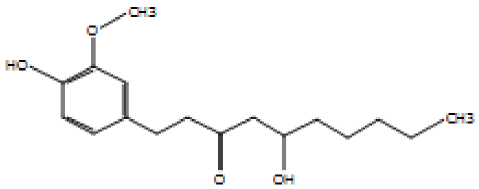

**Spectrum Source**	**Collision Energy**	**Ionization Mode**
Peak (27) in "+ TIC Scan"	0	Unspecified

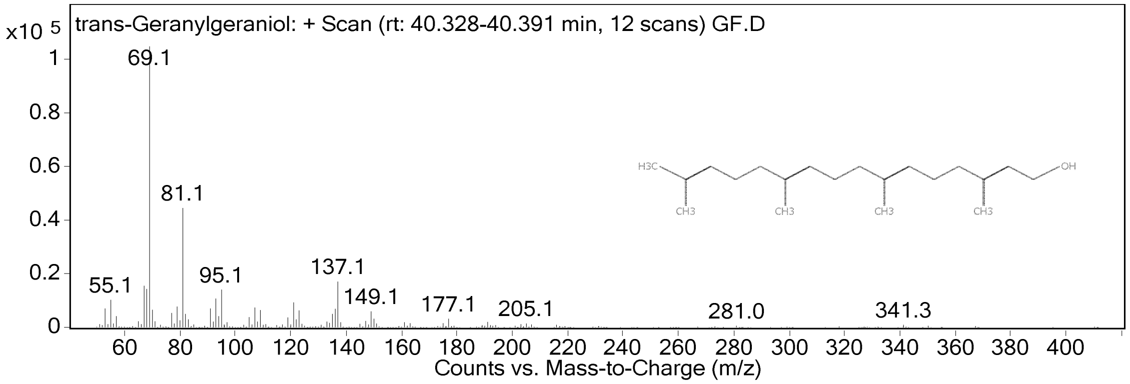

Peak List

m/z	Abund
53.1	7047.5
55.1	10257.4
67.1	15510.12
68.1	14285.79
69.1	104740.73
70.1	6515.98
77	5367.62
79.1	7682.51
81.1	44517.97
91.1	7047.55
93.1	10642.77
95.1	14046.41
107.1	7405.56
109.1	6390.29
121.1	9271.35
123.1	6322.89
135.1	4966.95
136.1	6885.22
137.1	17050.94
149.1	5956.21

Spectrum Structure

trans-Geranylgeraniol

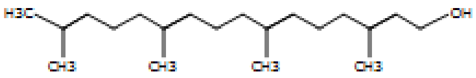

--- End Of Report ---
